# Introgression of a Danbaekkong high-protein allele across different genetic backgrounds in soybean

**DOI:** 10.3389/fpls.2023.1308731

**Published:** 2023-12-20

**Authors:** Renan Souza, M. A. Rouf Mian, Justin N. Vaughn, Zenglu Li

**Affiliations:** ^1^ Department of Crop and Soil Sciences, University of Georgia, Athens, GA, United States; ^2^ Soybean and Nitrogen Fixation Research Unit, United States Department of Agriculture - Agricultural Research Service (USDA-ARS), Raleigh, NC, United States; ^3^ Genomics and Bioinformatics Research Unit, United States Department of Agriculture - Agricultural Research Service (USDA-ARS), Athens, GA, United States

**Keywords:** soybean, seed protein, Danbaekkong, chromosome 20 QTL, multiparent populations, yield

## Abstract

Soybean meal is a major component of livestock feed due to its high content and quality of protein. Understanding the genetic control of protein is essential to develop new cultivars with improved meal protein. Previously, a genomic region on chromosome 20 significantly associated with elevated protein content was identified in the cultivar Danbaekkong. The present research aimed to introgress the Danbaekkong high-protein allele into elite lines with different genetic backgrounds by developing and deploying robust DNA markers. A multiparent population consisting of 10 F_5_-derived populations with a total of 1,115 recombinant inbred lines (RILs) was developed using “Benning HP” as the donor parent of the Danbaekkong high-protein allele. A new functional marker targeting the 321-bp insertion in the gene *Glyma.20g085100* was developed and used to track the Danbaekkong high-protein allele across the different populations and enable assessment of its effect and stability. Across all populations, the high-protein allele consistently increased the content, with an increase of 3.3% in seed protein. A total of 103 RILs were selected from the multiparent population for yield testing in five environments to assess the impact of the high-protein allele on yield and to enable the selection of new breeding lines with high protein and high yield. The results indicated that the high-protein allele impacts yield negatively in general; however, it is possible to select high-yielding lines with high protein content. An analysis of inheritance of the Chr 20 high-protein allele in Danbaekkong indicated that it originated from a *Glycine soja* line (PI 163453) and is the same as other *G. soja* lines studied. A survey of the distribution of the allele across 79 *G. soja* accessions and 35 *Glycine max* ancestors of North American soybean cultivars showed that the high-protein allele is present in all *G. soja* lines evaluated but not in any of the 35 North American soybean ancestors. These results demonstrate that *G. soja* accessions are a valuable source of favorable alleles for improvement of protein composition.

## 1 Introduction

Soybean [*Glycine max* (L.) Merr.] is one of the most important sources of protein and oil for direct and indirect human use. Soybean oil is omnipresent in the food industry, while soybean meal is the primary source of protein for livestock. Over the past 33 years, soybean yield in the United States increased 40.8%; however, the protein content went in an opposite direction, decreasing from 35.8% to 33.5% (Naeve and Miller-Garvin, 2021). Farmers and grain processors historically have had no incentive to produce and deliver soybeans with high protein and therefore no focus has been given in improving this seed component. The reduction in seed protein has negative effects on soybean value, as lower protein content makes it difficult to meet the requirements of the livestock industry for feed (Brumm and Hurburgh, 1990; de Borja Reis et al., 2020).

The genetic component is a major factor in the determination of seed composition in soybean. Lee et al. (2019) demonstrated the importance of the genetic factors for protein composition (heritability of 0.94) and confirmed the antagonist relationship between protein and oil (*r* = −0.75; *p* < 0.0001). More than 160 protein quantitative trait loci (QTLs) from 35 different studies have been reported (Patil et al., 2017) and one of these QTLs, located on chromosome (Chr) 20, has been repeatedly identified in several studies (Diers et al., 1992; Hwang et al., 2014; Vaughn et al., 2014; Warrington et al., 2015; Qi et al., 2016). This QTL has received the attention of many researchers because of its high additive effect and stability (Lestari et al., 2013). Warrington et al. (2015) demonstrated that this QTL explained 55% of the phenotypic variation of seed protein content in a bi-parental population derived from a cross of “Benning” (PI 595645) (Boerma et al., 1997) and Danbaekkong (PI 619083) (Kim et al., 1996). Danbaekkong is a South Korean cultivar that contributed to high protein content in the population (Warrington et al., 2015).

Despite the negative relationship of protein with oil and yield, there were reports on the feasibility of developing lines with increased protein content and high yield (Cober and Voldeng, 2000; Brzostowski et al., 2017). Prenger et al. (2019) developed Benning HP as a near-isogenic line (NIL) with a high-protein allele on Chr 20 by backcrossing an F_5_-derived line from Benning × Danbaekkong to the recurrent parent Benning. This line has high protein content and yield equivalent to the recurrent parent Benning, demonstrating that it is possible to mitigate the negative effects of the high-protein allele on yield with progeny selection. However, it is still not clear how the protein and yield relationship work in multiple genetic backgrounds.

A Chr 20 QTL for protein content was detected in the same location of previous mapping studies in a genome-wide association study (GWAS) with accessions from the USDA Soybean Germplasm Collection conducted by Vaughn et al. (2014). Bandillo et al. (2015) also analyzed 12,000 accessions from the same collection and identified a protein QTL in the same region. The GWAS hits in these studies were associated with the alleles frequently found in Korean accessions. Using the similar dataset, Patil et al. (2017) performed a genome-wide phylogenetic analysis comparing Danbaekkong, North American Soybean Ancestors (NASA), Asian landraces, and several *Glycine soja* lines. When all SoySNP50K SNPs were considered, Danbaekkong was clustered with the NASA; however, when SNPs in the range of 27–32 Mb on Chr 20 were analyzed, Danbaekkong was clustered separately from NASA. This result indicated that NASA likely have a different allele from Danbaekkong at the Chr 20 and introgression of the high-protein allele into elite soybean lines could improve the seed protein content.

Soybean accessions in the USDA Germplasm Collection have great variation for protein content, with accessions reaching up to 57% of seed protein (USDA, 2023). This resource can be tapped to increase the overall protein content and quality in soybean breeding programs. It has been observed that *G. max* cultivars developed in Asia, especially in South Korea, usually have a higher content of seed protein than those developed in other countries (Vaughn et al., 2014; Bandillo et al., 2015; Patil et al., 2017). It is likely a result of the historical breeding efforts in that region to focus on the improvement of seed composition for soy food products, such as tofu and soy sauce (Lee et al., 2015). Danbaekkong is a cultivar developed in South Korea based on the selection for seed yield, protein content, quality, and tofu yield (Kim et al., 1996). The Korean accessions with high protein content are an important source of genetic diversity that can be used in U.S. soybean breeding programs to improve nutritional composition.

Recently, a gene was identified underlying control of the protein QTL on Chr 20. Fliege et al. (2022) performed fine mapping in multiple populations using a *G. soja* line (PI 468916) as the QTL donor and narrowed the QTL interval to a region of 77.8 kb. In this interval, a 321-bp fragment was present in the 4th exon of the gene G*lyma.20g085100* in low-protein lines. Using an RNAi experiment, the authors demonstrated that the variation in *Glyma.20g085100* was responsible for the difference in protein content. Similarly, Goettel et al. (2022) indicated that *Glyma.20g085100* is the gene responsible for elevated protein at the Chr 20 QTL and soybean lines without the 321-bp insertion exhibit increased protein content, while the lines with the 321-bp insertion had low protein. The authors concluded that the insertion was likely caused by a transposable element, and during the domestication process, the insertion allele is fixed in most *G. max* lines.

In the present research, we aimed to validate the Chr 20 QTL from Danbaekkong for increased protein content, introgress the allele into a wide range of genetic backgrounds for protein improvement, and elucidate the inheritance of the Danbaekkong high-protein allele.

## 2 Materials and methods

### 2.1 Plant materials and population development

The population consisting of 140 recombinant inbred lines (RILs) derived from Benning × Danbaekkong originally used to map the Chr 20 QTL was analyzed to saturate the QTL region. The seeds, original phenotypic data, and genotypic data were obtained from Warrington et al. (2015). To enable identification of polymorphisms in the QTL region, seven soybean lines with high and low protein content were selected for genome sequencing (**Supplementary Table S1**). The elite parent Benning and the high-protein parent Danbaekkong (PI 619083) were sequenced together with one high-protein *G. soja* accession (PI 163453) and three high-protein *G. max* lines (PI 398589, PI 408012, and PI 602447) that have a haplotype in the QTL region similar to Danbaekkong. The sequence of the *G. soja* accession PI 468916 that was used in the original mapping study of the Chr 20 QTL by Diers et al. (1992) was obtained from Zhou et al. (2015) and Bayer et al. (2021).

A set of 10 populations was developed by crossing Benning HP with 10 elite lines in 2016 (**Supplementary Table S2**). Benning HP is a MG VII NIL of Benning (PI 595645), carrying the introgression of the Chr 20 high-protein allele from Danbaekkong (PI 619083) (Prenger et al., 2019). The populations have a structure of a nested association mapping population, where Benning HP is the hub parent (**Supplementary Figure S1**).

The F_1_ generation was grown in the University of Georgia (UGA) greenhouse in Athens, GA during the winter of 2016–2017. During the summer of 2017, the F_2_ generation was grown at the UGA Iron Horse Farm in Watkinsville, GA, and then two cycles of single seed descent advancement were conducted to advance the F_3_ and F_4_ generations during the winter of 2017–2018 in the Puerto Rican nursery. In 2018, the F_5_ generation was grown at the UGA Iron Horse Farm and plants from each population were harvested and threshed individually. In the summer of 2019, plant rows were grown in an unreplicated augmented design along with the parents and three commercial check cultivars AG5534, AG6534, and AG7934.

### 2.2 Whole genome re-sequencing

The lines selected for sequencing were grown in a greenhouse and leaf tissue was collected 3 weeks after planting. For each genotype, a bulked sample of 12 plants were collected and leaf tissue was lyophilized and ground. Genomic DNA was extracted using the GeneJet Plant Genomic DNA purification mini kit (Thermo Scientific, Boston, MA, USA) and 150-bp DNA fragments were sequenced with the NextSeq Sequencing instrument (Illumina, San Diego, CA). Adapters were removed from the raw Fastq files using Trimmomatic v0.36 (Bolger et al., 2014), and sequencing reads were mapped to the soybean genome Wm82.a2.v1 (https://data.jgi.doe.gov) with Bowtie2 v2.3.3.1 (Langmead and Salzberg, 2012). SNP and indel calls were performed with the GATK HaplotypeCaller software (McKenna et al., 2010) and variants were annotated with SNPeff version 4.3t (Cingolani et al., 2012). Variant visualization in the Chr 20 QTL region was performed with the Integrative Genomics Viewer (IGV - v2.9.5) (Robinson et al., 2011).

### 2.3 Marker design and genotyping

The RILs from the Benning × Danbaekkong population were planted in the greenhouse and DNA extraction was performed on leaf tissue using the CTAB method (Keim et al., 1988). For the multiparent population, DNA was extracted from seed samples from all 1115 RILs in the 10 populations with a modified Edwards extraction (Edwards et al., 1991). KASP (LGC, Hoddesdon, UK) and TaqMan assays (Applied Biosystems, Foster City, CA) were designed using Geneious Primer version 2021.2 based on polymorphisms present in the QTL region identified from the SoySNP50K data (Song et al., 2013) and whole genome sequence of the seven sequenced soybean lines (Danbaekkong, Benning, PI 163453, PI 398589, PI 408012, PI 602447, and PI 468916) (**Supplementary Tables S3**–**S5**). The gene-specific marker GSM1252 targeting the 321-bp insertion at the gene *Glyma.20g085100* was designed based on information previously published by Fliege et al. (2022) and Goettel et al. (2022).

KASP reactions were performed in a 4-μL volume with 2 μL of master mix (1.97 μl of KASP 2X and 0.053 μL of primers) and 2 μL of 10–20 ng/μL genomic DNA. Similarly, TaqMan reactions were also conducted in a 4-μL volume including 2 μL of master mix (2 μL of TaqMan Universal Master Mix II and 0.2 μL of 5X Custom TaqMan SNP Genotyping Assay) and 2 μL of 10–20 ng/μL genomic DNA. PCR was performed in the BioRad C1000 Touch Thermal Cycler and PCR plates were read in either LightCycler^®^ 480 (Roche, Germany) or TECAN infinite M200 microplate reader (Tecan US, Inc, Durham, NC) using the software KlusterCaller (version 2.24.0.11, LGC Genomics). Cycling conditions for the KASP assays were 15 min at 94°C, 10 cycles of 15 s at 94°C and 1 min at 65°C and 30 cycles of 20 s at 94°C and 1 min at 57°C. Cycling conditions for the TaqMan followed a modified touchdown PCR with an initial 10 min at 95°C, 10 cycles of 20 s at 95°C and 1 min at 71°C, decreasing 0.5°C each cycle, and 30 cycles of 15 s at 92°C and 1 min at 58°C.

### 2.4 Diversity panel

To analyze the distribution of the Chr 20 high-protein QTLs, 35 NASA (Gizlice et al., 1994) and 79 diverse *G. soja* accessions (La et al., 2019) were genotyped using the gene-specific TaqMan marker GSM1252. The 35 *G. max* soybean ancestors contributed 95% of the genes found in modern soybean cultivars (Gizlice et al., 1994) and the 79 *G. soja* lines are a core set that represent the genetic diversity within the entire USDA *G. soja* Collection (La et al., 2019). These accessions were planted in the greenhouse and leaf tissue was collected 2 weeks after planting. DNA extraction was performed with the CTAB method (Keim et al., 1988).

The seed composition of the 79 *G. soja* accessions was obtained from La et al. (2019) and the data for 25 of 35 North American Soybean ancestors were collected with the Near-Infrared Spectroscopy Perten DA 7250 Analyzer (PerkinElmer Inc., Waltham, MA, USA) from seeds harvested in the USDA winter nursery in Puerto Rico in 2018. The phenotypes of the remaining 10 accessions were retrieved from USDA GRIN (https://npgsweb.ars-grin.gov/gringlobal/).

Another panel of 35 *G. soja* lines was assembled to compare the genome sequence variation at the gene level and survey the distribution of the high-protein allele. The raw sequencing data were generated in previous studies (Bayer et al., 2021; Valliyodan et al., 2021) and are available at the Short Read Archive (SRA) database at NCBI (www.ncbi.nlm.nih.gov). Adapters were removed from the raw Fastq files using Trimmomatic v0.36 (Bolger et al., 2014) and sequencing reads were mapped to the soybean genome Wm82.a2.v1 with Bowtie2 v2.3.3.1 (Langmead and Salzberg, 2012).

### 2.5 Yield trials of selected RILs

To understand the effects of the Danbaekkong high-protein allele on yield in different genetic backgrounds, a set of RILs from the multiparent populations with high and normal protein content were selected for evaluation in yield trials. A total of 103 lines were planted in three locations in 2020 and 2021. In 2020, all the lines were grown in a randomized complete block design with two replications per location and each line was planted in a 2-row plot with a length of 4.9 m spaced by 76.2 cm and a planting density of 27 seeds m^–1^. A total of 46 lines were selected based on yield and agronomic performance and grown in 2021 in a randomized complete block design with three replications in a 4-row plot with the same plot length and row spacing. The commercial cultivars AG 64X8RR2X, AG 74X8RR2X, and AGS 738RR were used as checks across the different environments. Agronomic practices followed the recommended guidance for soybean production in Georgia (Bryant, 2020). All plots were end-trimmed before harvest to avoid edge effect, resulting in a length of 3.7 m. The two center rows were harvested, and weight and moisture were measured on combines. Approximately 200 seeds were sampled from each plot for seed composition analysis.

### 2.6 Seed composition analysis

The contents of protein and oil were determined using the NIR Perten DA 7250 Analyzer (PerkinElmer Inc., Waltham, MA, USA) and the instrument was calibrated by the manufacturer using thousands of samples with known seed composition values for whole seed and ground seed samples. Seed composition was reported on a dry matter basis. Analysis of the multiparent population was performed on the seeds from single plants in 2018 and from the plant rows in 2019. For the yield trials in 2020 and 2021, samples of 200 seeds were obtained from each plot.

### 2.7 Statistical and QTL analyses

Phenotypic and genotypic data were analyzed in RStudio (R version 3.4.4) using the packages lme4 (Bates et al., 2015) and BreedR (Muñoz and Rodriguez, 2020), and data visualization was created with ggplot2 (Wickham, 2016). The phenotypic values for the QTL analysis of the multiparent population were calculated by fitting a model with the subpopulation and year effects as fixed and the genotype effect as random. For the data from the Benning × Danbaekkong RIL population, best linear unbiased predictions (BLUPs) were obtained by fitting a model with the environment (location + year) as a fixed effect and genotype and replication as random effects. Analysis of the phenotypic data from yield trials with the 103 selected breeding lines was performed by fitting a model with the QTL within each subpopulation as a fixed effect, and genotype, environment, and replication as random effects.

To saturate the QTL region identified in the Benning × Danbaekkong RIL population, additional markers were developed in the QTL interval based on comparison of the sequencing data from the seven sequenced genotypes. Linkage map construction and QTL analysis were performed with the R package R/qtl (Broman et al., 2003). Associations between markers and protein content were established with a regression function using a LOD significance threshold determined by 1,000 permutations. Recombination distances were calculated using Kosambi’s mapping function with simple interval and composite interval mapping methods in the QTL position estimation. To understand the effects of the QTL in a broad genetic background, a multiparent population QTL analysis was performed using an R package mppR (Garin et al., 2020). In each round of mapping, the population was randomly partitioned into five subsets and one of the subsets was used for validation of the parameters calculated in the other four subsets. Composite interval mapping was performed in each subset 100 times and the QTL position was determined by the location of the most significant marker across all iterations.

## 3 Results

### 3.1 Danbaekkong high-protein allele

**Figure 1:**
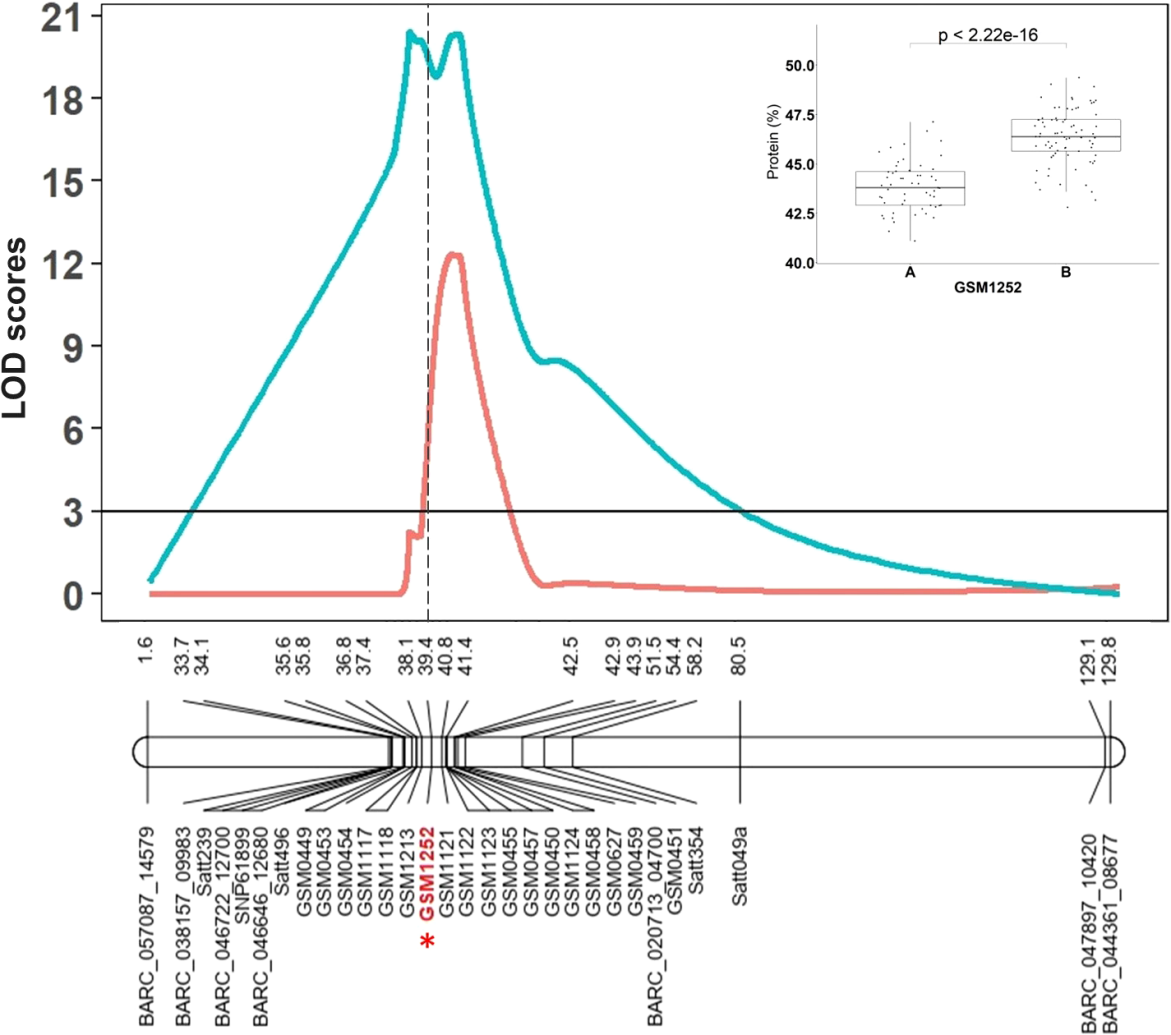
The Chr 20 QTL region identified by Warrington et al. (2015) in the RIL population derived from Benning × Danbaekkong and saturated with additional KASP markers and the gene-specific TaqMan marker GSM1252 is indicated in red with an asterisk. Red lines indicate Composite Interval Mapping and blue lines indicate Simple Interval Mapping. Marker distances are given in centimorgan (cM). Additional information about the markers is presented in **Supplementary Tables S3**–**S5**.

The RIL population derived from the Benning × Danbaekkong cross (*N* = 140) was genotyped with the Chr 20 QTL flanking markers previously used by Warrington et al. (2015) and 17 new additional markers designed based on variants found in the comparison of the seven sequenced lines. The markers were combined to saturate the Chr 20 QTL region and one of the markers used (GSM1252) specifically targeted the 321-bp insertion in the gene *Glyma.20g085100* identified by Fliege et al. (2022). The QTL interval was identified in a genomic region between 27.7 and 33.0 Mb across all environments tested and the marker GSM1252 designed from the gene *Glyma.20g085100* was one of the most significant markers across the environments (**Figure 1**). In this population, the QTL explained 47.5% of the phenotypic variation and had an additive effect of 1.3% in the protein content. The homozygous RILs for the low-protein allele at the GSM1252 locus had an average protein content of 43.8%, while the lines with the homozygous high-protein allele had a protein content of 46.4%.

**Figure 2:**
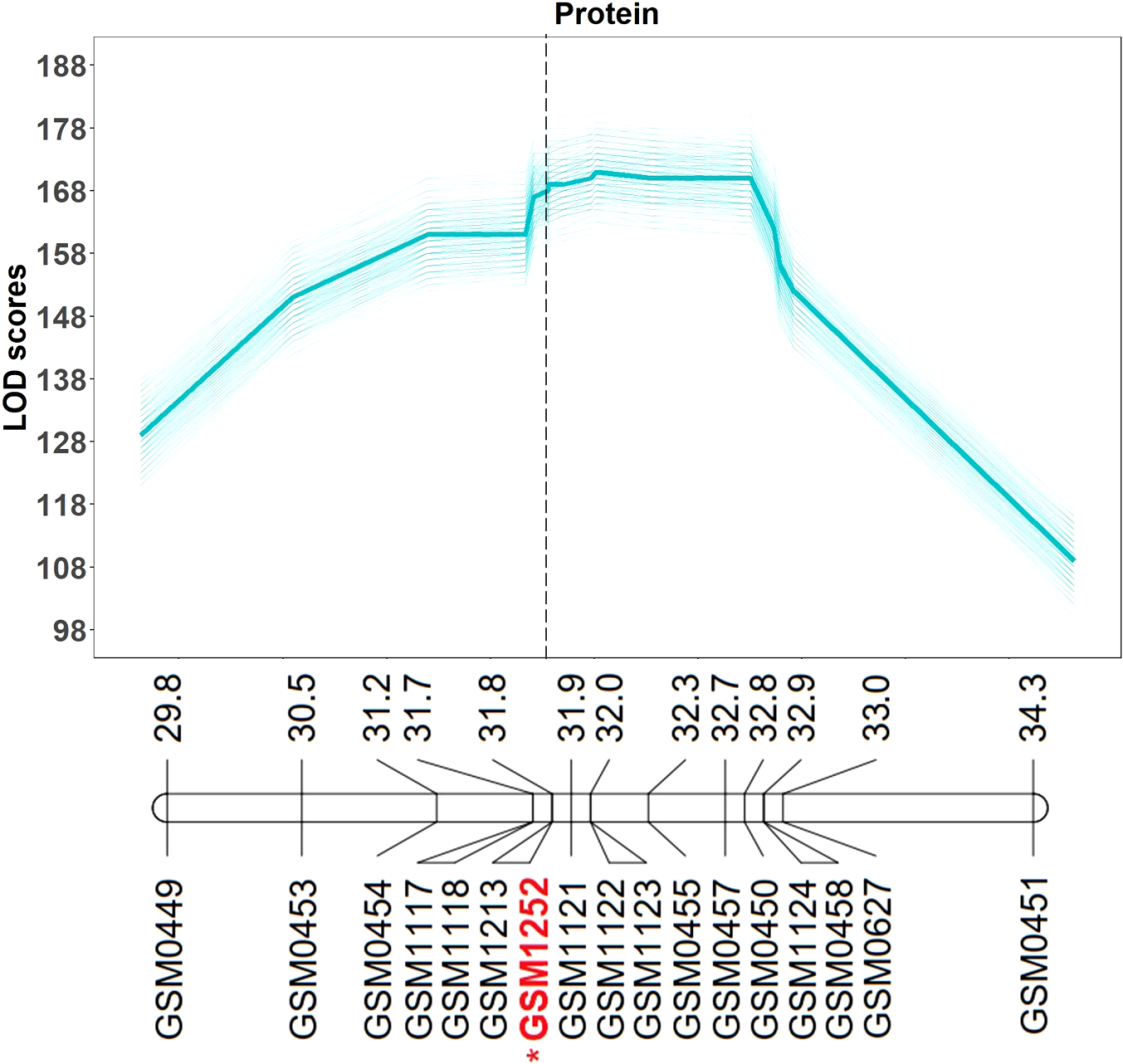
Multiparent population QTL analysis for seed protein and oil content. QTL analysis was performed 500 times (5 random subsets with 100 replications) using the composite interval mapping function. The average LOD value of all values is indicated in the bold line. Mapping was performed with the KASP markers and the gene-specific TaqMan marker GSM1252 indicated in red with an asterisk. Marker position is given in Mb based on Wm82.a2.v1. Additional information about the markers is presented in **Supplementary Tables S3**–**S5**.

**Figure 3:**
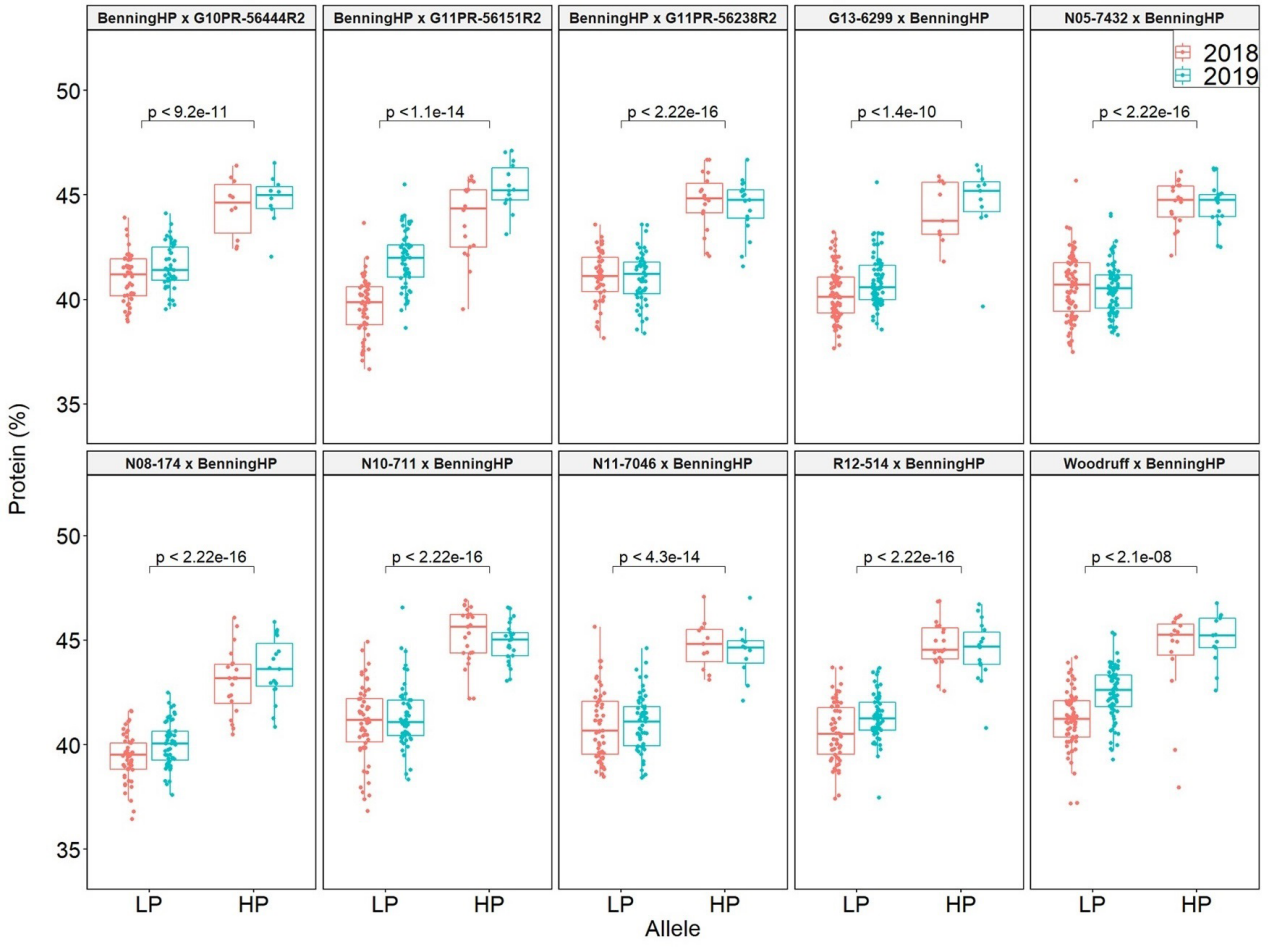
Effects of the Danbaekkong Chr 20 high-protein allele on seed protein in 10 RIL populations evaluated in 2018–2019. The *X* axis indicates the allele at the *Glyma.20g085100* (GSM1252). HP and LP represent the high- and low-protein alleles as indicated by the gene-specific marker GSM1252, respectively. Protein content is on a dry matter basis.

**Figure 4:**
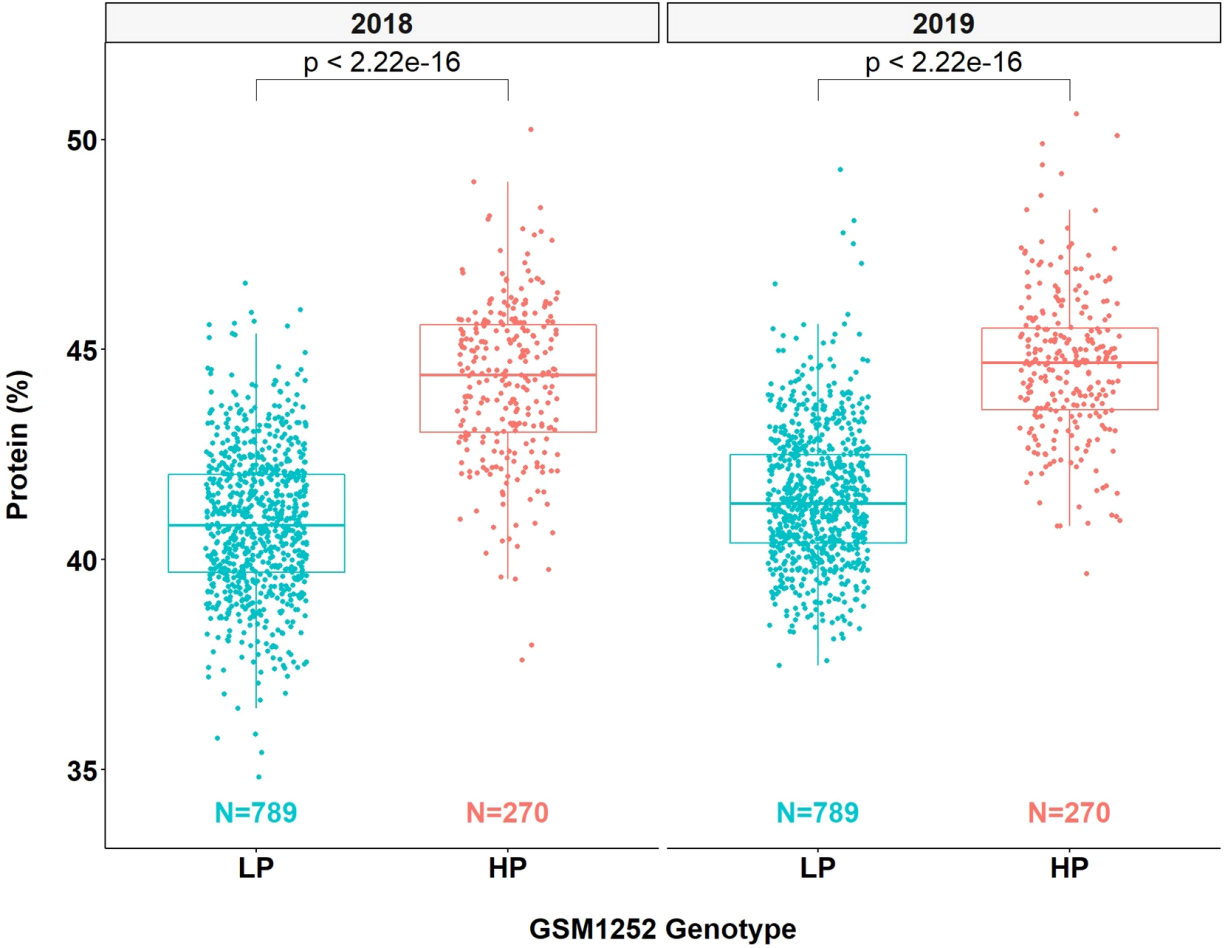
Effects of different alleles at *Glyma.20g085100* on protein content across the multiparent RIL populations. HP indicates the high-protein allele and LP indicates the low-protein allele at GSM1252.

QTL mapping was also performed in the multiparent population and the QTL region was identified to the interval between 31.8 and 32.2 Mb (markers GSM1252 and GSM0455). This region is located within the QTL interval identified in the analysis of the Benning × Danbaekkong RIL population (**Figure 2**). After the estimation of the QTL parameters, an association analysis between the *Glyma.20g85100* marker (GSM1252) and the content of protein and oil was performed using the lines in the multiparent population. The high-protein allele from Danbaekkong was associated with an increase in the protein of 3.3% on average (ranging from 2.6% to 3.7%) across all populations. The highest increase in protein content was observed in population G13-6299 × Benning HP, with protein content going from 40.8% to 44.5%. The highest average value of protein obtained was in the population Woodruff × Benning HP with lines carrying the high-protein allele reaching 45.4% (**Figures 3**, **4**; **Supplementary Table S6**).

The increase in the protein content was accompanied by a reduction in oil content in all populations, ranging from a reduction of 1.4% in Benning HP × G10PR-56444R2 to 2.0% in N10-711 × Benning HP and Benning HP × G11PR-56238R2. On average, for every 1.8% increase in protein, there was a decrease of 1% in oil. The populations N08-174 × Benning HP and Benning HP × G10PR-56444R2 had an average oil content ≥20% and protein content ≥43.5%, demonstrating the possibility of having high protein and oil above 20% (**Supplementary Table S6**).

The fact that Benning HP was used either as a male or female parent in the multiparent population enabled evaluation of any maternal effect of the Danbaekkong high-protein allele. It was observed that the Danbaekkong high-protein allele increased the protein in a similar magnitude having the Benning HP as the female (44.5%) or the male (44.3%) parent in the cross (**Supplementary Table S6**).

### 3.2 Effects of the Danbaekkong high-protein allele on yield

To assess the effects of the protein QTL on yield, 103 RILs were selected from the multiparent population based on agronomic performance and visual assessment of plant appearance, lodging, and maturity to enter the 2020 and 2021 yield trials. Population N10-711 × Benning HP had the highest number of lines in the trials (27 in 2020 and 13 in 2021), while Benning HP × G11PR-56238R2 had the lowest number, with three lines in the yield trials. Overall, all pedigrees had lines with the high-protein allele or low-protein allele variant evaluated in both years, except for the Benning HP × G11PR-56238R2 population, which was evaluated only in 2020 and Benning HP × G10PR-56444R2 did not have lines with the high-protein allele tested. Having lines with and without the Danbaekkong high-protein allele evaluated in yield trials in 9 of the 10 pedigrees enabled a comparison of the effects of the increased protein content on yield in multiple genetic backgrounds.

**Figure 5:**
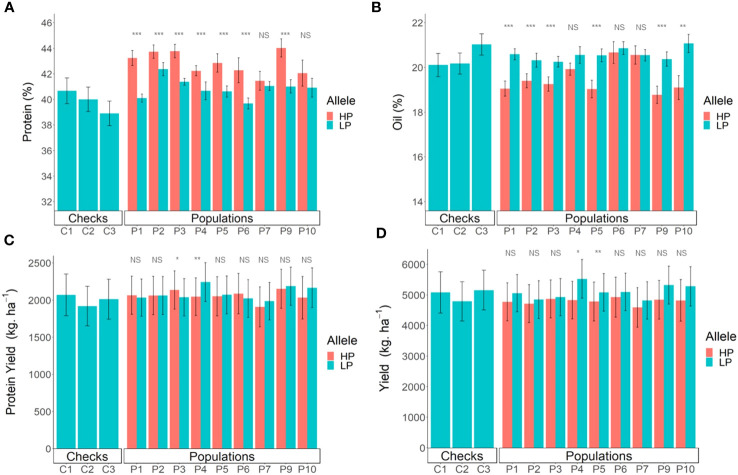
Comparison of breeding lines with and without the Danbaekkong Chr 20 high-protein allele in each population. Red indicates lines with the high-protein allele (HP) and blue indicates lines with the low-protein allele (LP). **(A)** Comparison of the protein content, **(B)** Oil content, **(C)** Production of protein per hectare, and **(D)** Seed yield. A total of 103 RILs were evaluated in five environments (Athens 2020 and 2021, Plains 2020 and 2021, and Tifton 2021) with two to three replications per environment. Error bar indicates standard error. Checks C1, C2, and C3 correspond to AG 64X8RR2X, AG 74X8RR2X, and AGS 738RR, respectively. *, **, and *** indicate significance at the 0.05, 0.01, and 0.001 probability level and NS indicates not significant.

In the yield trials, the lines carrying the high-protein allele had a consistently higher protein content across all the populations, with an average increase of 2.0% in protein content. The only exception was population R12-514 × Benning HP, in which lines with the high-protein allele in the population did not have a significant increase in protein content. Population G13-6299 × Benning HP had the highest increase in protein, from 40.1% to 43.2% and population N10-711 × Benning HP had the highest average value of protein, with 43.8% (**Figure 5A**). The oil content had an overall reduction of 1%, but variation was observed across the different populations, ranging from a 2% reduction in Benning HP × G11PR-56238R2 to no detectable reduction in R12-514 × Benning HP (**Figure 5B**). In the comparison of the protein production per hectare, the populations also had different performances. Lines with the high-protein allele from the population N10-711 × Benning HP had an increase of 94 kg ha^−1^ in protein production, but in the population N05-7432 × Benning HP, the lines with the high-protein allele had a decrease of 218 kg ha^−1^. When considering the performance of all populations together, there was no difference (*p* = 0.41) in the protein production per hectare in the lines with or without the Danbaekkong high-protein allele, 2,048 vs. 2,080 kg ha^−1^, respectively (**Figure 5C**; **Supplementary Table S7**).

Overall, the high protein negatively impacts the yields, with an average reduction of 313 kg ha^−1^. However, there was variation across the different populations, with population N05-7432 × Benning HP having a yield reduction of 719 kg ha^−1^ to the population N10-711 × Benning HP with a yield reduction of only 55 kg ha^−1^ in the lines with the high-protein allele. Of the 103 lines evaluated, 20 lines from different populations had yield similar to or higher than the commercial check AGS 738RR, and 14 of these lines had a protein content higher than 40% (**Figure 5D**; **Supplementary Table S8**). The line G19-11395 from population N05-7432 × Benning HP did not have the high-protein allele but stood out with the highest overall yield, 5,880 kg ha^−1^, 13.8% higher but not significantly different from AGS-738RR. The line G19-11191 from population Woodruff × Benning HP was the only line carrying the high-protein allele (43.6% protein) that had yield comparable to the AGS 738RR (100.4%), with 5,189 kg ha^−1^. Three other lines, G19-11422 (N05-7432 × Benning HP), G19-11111 (G13-6299 × Benning HP), and G19-2139R2 (Benning HP × G11PR-56151R2), carrying the high-protein allele at GSM1252, had a protein content exceeding 43% and yielded >95% of AGS 738RR (**Supplementary Table S8**). These results exemplify the possibility of combining high yield with improved seed composition.

### 3.3 Effect of maturity on seed protein

The association between maturity and the high-protein alleles was evaluated across the different pedigrees in the multiparent population. Nine out of 10 populations studied had the lines carrying the high-protein allele reaching maturity earlier than those with normal protein. Overall, high-protein lines reached maturity 3.5 days earlier than those with low protein (**Table 1**). The population with the biggest difference was N05-7432 × Benning HP, in which the lines having the high-protein allele matured 6.1 days earlier than those with the low-protein allele. On the other hand, there was no significant difference in maturity between lines with the high-protein allele and those with the low-protein allele in the population N08-174 × Benning HP.

**Table 1:** Effects of the high-protein QTL on maturity.

Population	Pedigree	Maturity†		Difference
		HP lines	LP lines	
P1	G13-6299 × Benning HP	52.8	55.3	2.5*
P2	Woodruff × Benning HP	53.0	55.5	2.5***
P3	N10-711 × Benning HP	47.8	52.3	4.5***
P4	N05-7432 × Benning HP	51.1	57.2	6.1***
P5	N11-7046 × Benning HP	48.7	52.0	3.3**
P6	N08-174 × Benning HP	41.4	41.9	0.5^ns^
P7	R12-514 × Benning HP	40.7	44.2	3.4*
P8	Benning HP × G10PR-56444R2	52.4	55.3	2.9***
P9	Benning HP × G11PR-56151R2	48.7	54.3	5.6***
P10	Benning HP × G11PR-56238R2	48.4	53.5	5.1***

HP indicates the high-protein allele and LP indicates the low-protein allele at GSM1252.

*, **, and *** indicated significant differences at the 0.05, 0.01, and 0.001 probability level, respectively.

† Maturity is indicated as days after August 31.

### 3.4 Distribution of the Chr 20 high-protein allele among the soybean ancestors and *G. soja* lines

**Figure 6:**
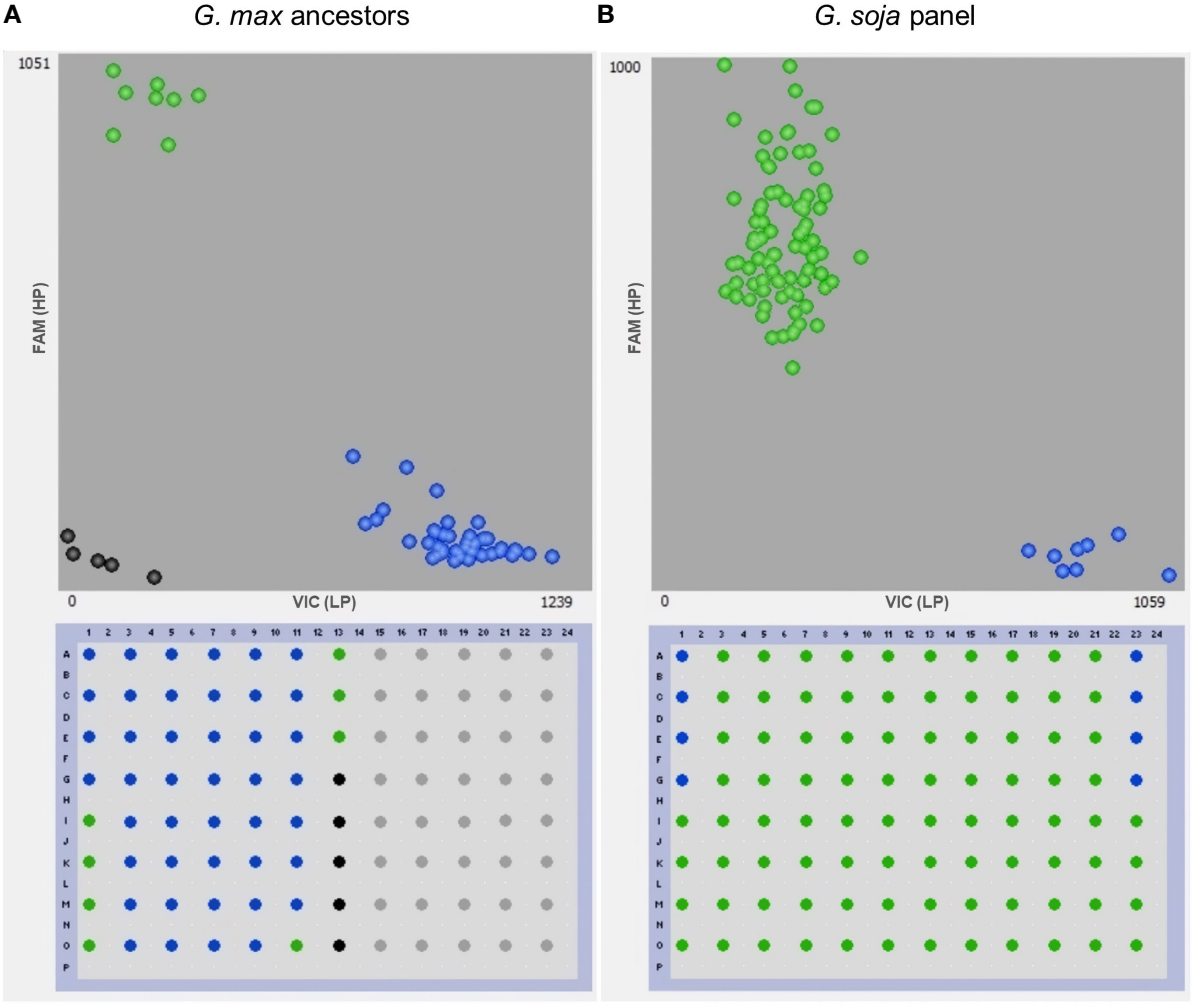
Genotyping with the gene-specific TaqMan marker GSM1252. **(A)** 35 North America *Glycine max* ancestors and **(B)** 79 *Glycine soja* accessions. Benning and Benning HP were used as controls four times on each plate for the low-protein (LP) and the high-protein allele (HP), respectively. Points in green, blue, and black colors denote the high-protein allele, low-protein allele, and no template control, respectively.

The presence of the Danbaekkong high-protein allele was surveyed using the gene-specific TaqMan marker GSM1252 in a panel of 35 *G. max* ancestral lines that contributed 95% of the genes found in modern soybean cultivars (Gizlice et al., 1994). These lines provided a good opportunity to understand the distribution of the high-protein allele in the North American soybean breeding pool. The results indicated that all 35 *G. max* ancestors have the low-protein allele at the gene *Glyma.20g085100*, and the average protein content was 41.6% (ranging from 38.1% to 45.7%) (**Table 2**; **Figure 6**).

**Table 2:** Distribution of the low-protein allele (321-bp insertion) among North American soybean ancestral lines as defined by Gizlice et al. (1994).

	ID	Origin	MG	Protein (%)	Oil (%)	GSM1252^†^
1	FC 31745	–	VI	40.2	21.5	LP
2	FC 33243	–	IV	38.1	22.5	LP
3	PI 180501^‡^	Germany	0	39.1	21.3	LP
4	PI 240664^‡^	Philippines	X	44.8	21.1	LP
5	PI 360955B^‡^	Sweden	0	42.7	18.2	LP
6	PI 438471	Sweden	0	38.2	20.3	LP
7	PI 438477	Sweden	0	39.6	19.7	LP
8	PI 548298	China	III	43.0	19.9	LP
9	PI 548302	Japan	II	42.2	17.8	LP
10	PI 548311^‡^	Canada	0	42.0	20.4	LP
11	PI 548318^‡^	China	III	39.1	21.6	LP
12	PI 548325	Russia	0	41.5	19.7	LP
13	PI 548348	China	III	41.5	20.0	LP
14	PI 548352^‡^	North Korea	III	41.4	19	LP
15	PI 548356^‡^	North Korea	II	41.4	19.9	LP
16	PI 548360	North Korea	II	39.7	21.4	LP
17	PI 548362	United States	III	38.4	22.9	LP
18	PI 548379	China	0	38.4	20.9	LP
19	PI 548382^‡^	–	0	43.1	17.6	LP
20	PI 548391	China	II	43.0	20.3	LP
21	PI 548402^‡^	China	IV	38.2	18.5	LP
22	PI 548406	China	II	41.6	19.0	LP
23	PI 548438	North Korea	VI	44.7	19.2	LP
24	PI 548445	China	VII	45.7	19.0	LP
25	PI 548456	North Korea	VI	41.0	19.1	LP
26	PI 548461	United States	VIII	40.5	22.5	LP
27	PI 548477	United States	VI	42.9	20.2	LP
28	PI 548484	North Korea	VI	42.1	20.2	LP
29	PI 548485	China	VII	42.1	20.7	LP
30	PI 548488	China	V	43.8	18.9	LP
31	PI 548603	United States	IV	40.5	21.9	LP
32	PI 548657	United States	VII	40.3	21.9	LP
33	PI 71506	China	IV	41.0	22.6	LP
34	PI 80837^‡^	Japan	IV	42.4	18.2	LP
35	PI 88788	China	III	43.4	15.7	LP
	Benning ^§^	United States	VII	41.9	21.3	LP
	Benning HP ^§^	United States	VII	45.6	19.0	HP
	Danbaekkong^¶^	South Korea	V	48.0	18.5	HP

Protein and oil analyzed with near-infrared (NIR) spectroscopy using a sample of approximately 200 seeds harvested in 2018.

Benning, Benning HP, and Danbaekkong are controls.

† GSM1252 indicates the presence of the high-protein allele (HP) or the low-protein allele (LP).

‡ Protein and oil content retrieved from GRIN. https://npgsweb.ars-grin.gov/gringlobal/.

§ Benning and Benning HP values are averages from 3 years of tests (2019, 2020, and 2021).

¶ Danbaekkong value is the average from 2 years of tests (2017 and 2021).

Another analysis was performed to study the distribution of the high-protein allele across *G. soja* accessions. A panel of 79 diverse *G. soja* that represent the genetic diversity in USDA Soybean Germplasm Collection was surveyed (La et al., 2019). All the *G. soja* lines evaluated presented the high-protein allele on the Chr 20 and had an average protein content of 44.4% (ranging from 39.8% to 49.4%) (**Table 3**; **Figure 6**). To confirm the presence of the high-protein allele in *G. soja*, the sequence of 35 accessions that have not been studied previously was analyzed for the presence of the insertion in *Glyma.20g085100*. Confirming the previous results, all *G. soja* lines evaluated have the high-protein allelic variant (**Supplementary Table S9**).

**Table 3:** Distribution of the high-protein allele among the USDA *Glycine soja* core set as defined by La et al. (2019).

	Name	Origin	MG	Protein (%)	Oil (%)	GSM1252^†^
1	PI 101404A	China	II	45.7	16.2	HP
2	PI 339871A	South Korea	V	42.9	16.6	HP
3	PI 342622A	Russia	I	43.7	16.1	HP
4	PI 366122	Japan	IV	44.1	16.6	HP
5	PI 378683	Japan	VI	46.7	16.4	HP
6	PI 378684B	Japan	VI	47.3	16.0	HP
7	PI 378686B	Japan	VI	46.0	16.3	HP
8	PI 378690	Japan	VII	45.3	16.3	HP
9	PI 378696B	Japan	VI	43.7	16.7	HP
10	PI 378697A	Japan	V	44.5	16.5	HP
11	PI 407020	Japan	V	44.0	16.8	HP
12	PI 407038	Japan	V	45.4	16.5	HP
13	PI 407042	Japan	V	44.9	16.3	HP
14	PI 407052	Japan	V	46.8	16.1	HP
15	PI 407059	Japan	–	46.7	16.1	HP
16	PI 407085	Japan	VI	44.8	16.5	HP
17	PI 407096	Japan	VII	47.2	16.3	HP
18	PI 407156	Japan	VI	44.7	16.5	HP
19	PI 407157	Japan	VI	47.8	16.3	HP
20	PI 407171	South Korea	IV	43.8	16.4	HP
21	PI 407179	South Korea	V	44.4	16.8	HP
22	PI 407191	South Korea	V	46.2	16.5	HP
23	PI 407195	South Korea	IV	44.4	16.5	HP
24	PI 407206	South Korea	V	46.4	16.3	HP
25	PI 407214	South Korea	V	46.7	16.4	HP
26	PI 407228	South Korea	V	49.5	15.8	HP
27	PI 407231	South Korea	V	44.4	16.5	HP
28	PI 407240	South Korea	V	46.3	16.5	HP
29	PI 407248	South Korea	V	44.6	16.6	HP
30	PI 407287	Japan	V	45.6	16.3	HP
31	PI 407300	China	V	46.1	16.2	HP
32	PI 407314	South Korea	V	44.2	16.9	HP
33	PI 424004B	South Korea	II	43.6	16.5	HP
34	PI 424007	South Korea	V	42.3	16.8	HP
35	PI 424025B	South Korea	V	46.3	16.4	HP
36	PI 424035	South Korea	V	43.3	16.7	HP
37	PI 424045	South Korea	V	42.6	16.5	HP
38	PI 424070B	South Korea	V	43.3	16.5	HP
39	PI 424082	South Korea	V	44.1	16.1	HP
40	PI 424083A	South Korea	V	45.4	16.4	HP
41	PI 424102A	South Korea	V	43.6	16.5	HP
42	PI 424116	South Korea	IV	43.7	16.6	HP
43	PI 424123	South Korea	V	44.0	16.1	HP
44	PI 447003A	China	0	43.8	16.8	HP
45	PI 458536	China	0	48.3	16.3	HP
46	PI 464890B	China	I	47.2	16.2	HP
47	PI 479746B	China	II	46.6	16.1	HP
48	PI 479751	China	III	43.7	16.8	HP
49	PI 479752	China	I	41.2	16.4	HP
50	PI 479768	China	0	44.8	16.4	HP
51	PI 483466	China	V	43.9	16.2	HP
52	PI 507618	Japan	V	44.1	16.4	HP
53	PI 507624	Japan	VII	44.6	16.4	HP
54	PI 507641	Japan	V	45.9	16.6	HP
55	PI 507656	Japan	VII	45.9	16.3	HP
56	PI 507761	Russia	I	42.4	16.4	HP
57	PI 522209B	Russia	II	43.2	16.4	HP
58	PI 522226	Russia	000	43.3	16.3	HP
59	PI 522233	Russia	I	44.3	16.1	HP
60	PI 522235B	Russia	I	41.6	16.2	HP
61	PI 549032	China	III	44.0	15.9	HP
62	PI 549046	China	III	39.9	17.1	HP
63	PI 549048	China	III	41.0	17.6	HP
64	PI 562547	South Korea	V	41.2	16.5	HP
65	PI 562551	South Korea	V	43.9	16.5	HP
66	PI 562553	South Korea	V	47.4	16.3	HP
67	PI 562561	South Korea	V	47.1	16.0	HP
68	PI 562565	South Korea	IV	43.2	16.4	HP
69	PI 593983	Japan	III	45.0	16.7	HP
70	PI 597448D	China	0	45.2	16.2	HP
71	PI 597458C	China	V	43.5	17.2	HP
72	PI 597460A	China	IV	42.9	16.8	HP
73	PI 597461B	China	V	39.8	17.5	HP
74	PI 597462B	China	IV	42.5	17.1	HP
75	PI 639586	Russia	–	42.0	17.0	HP
76	PI 639588B	Russia	–	41.8	17.1	HP
77	PI 639621	Russia	–	41.8	17.1	HP
78	PI 639623A	Russia	–	44.2	16.5	HP
79	PI 639635	Russia	–	43.3	16.4	HP
	PI 163453	China	VI	44.7	12.0	HP
	PI 468916	China	III	44.0	10.1	HP
	Benning^‡^	United States	VII	41.9	21.3	LP
	Benning HP^‡^	United States	VII	45.6	19.0	HP
	Danbaekkong^§^	South Korea	V	48.0	18.5	HP

Protein and oil contents for *G. soja* accessions were obtained from La et al. (2019).

All *G. soja* accessions have black seed coat color.

Benning, Benning HP, and Danbaekkong are controls. PI 163453 is the *G. soja* ancestor of Danbaekkong and PI 468916 is the *G. soja* used in Fliege et al. (2022).

† GSM1252 indicates the presence of the high-protein allele (HP) or the low-protein allele (LP).

‡ Benning and Benning HP values are averages from 3 years of tests (2019, 2020, and 2021).

§ Danbaekkong value is the average from 2 years of tests (2017 and 2021).

## 4 Discussion

### 4.1 Danbaekkong high-protein allele

Using new molecular markers positioned in the interval where the protein QTL has been repeatedly identified (29.8 to 34.3 Mb), genotyping was performed in the Benning × Danbaekkong RIL population (*N* = 140) and in a multiparent population (*N* = 1,115). Of these markers, GSM1252 was developed based on previous research that identified *Glyma.20g085100* controlling the protein at the Chr 20 QTL (Fliege et al., 2022; Goettel et al., 2022). GSM1252 was developed as a TaqMan marker with one probe targeting the flanking regions of the insertion aiming to capture the alleles without the 321-bp insertion and another probe that binds to a fragment of the insertion and the right flanking site (**Supplementary Figure S2**). Overall, the marker exhibited a good performance in separating the lines with and without the insertion and it is a useful tool to select lines for high protein. The QTL analysis confirmed the variation in *Glyma.20g085100* to be associated with protein content in the populations derived from Danbaekkong. However, instead of GSM1252, marker GSM1122 was the most significant marker at the locus. This can be attributed to the fact that Chr 20 QTL is a region of strong linkage disequilibrium (Vaughn et al., 2014). The data analysis in Benning × Danbaekkong and the multiparent population indicated a confidence interval of 503,806 bp between the markers GSM1252 and GSM0455 (31,778,817–32,282,623 bp) (Wm82.a2.v1). This region overlaps perfectly with previously published mapping work that identified the Chr 20 QTL (Bolon et al., 2010; Vaughn et al., 2014; Warrington et al., 2014; Lee et al., 2019; Wang et al., 2021). In the analysis of the multiparent mapping population, the flat QTL peak in the region between 31.8 and 32.8 Mb indicated that this genomic region has a large linkage disequilibrium block.

To elucidate the origins of the Danbaekkong high-protein allele, an analysis of the Danbaekkong pedigree was conducted. One of the Danbaekkong’s parents is the cultivar Dongsan 69 from South Korea and the pedigree of this cultivar is unknown since no release information is available. The other parent is D76-8070, which is an MG V line developed by Edgar Hartwig in his effort to breed soybean cultivars with increased protein content (Hartwig, 1990). D76-8070 was developed through the selection of progeny from multiple crosses (“Hill” × “Sioux”, FC 31745 × D49-2510, Hill × PI 96983, and D49-24914 × PI 163453). The progeny from each of these crosses were selected for disease resistance, agronomic traits, and high protein content (>45%) and the selected lines were intercrossed to develop D76-8070 (**Supplementary Figure S3**). PI 163453 is the only *G. soja* line present in the pedigree of D76-8070 and was hypothesized as the origin for the high-protein QTL. To verify this hypothesis, the haplotypes of PI 163453 and Danbaekkong were compared using the 6,353 SNPs between 30 and 34 Mb on Chr 20 called from the sequencing data. The genetic similarity analysis showed that the Danbaekkong haplotype at the Chr 20 QTL region is 99.95% identical to PI 163453 (**Supplementary Table S10**). To confirm the inheritance of the protein QTL, D76-8070 was also genotyped with GSM1252 and the results indicated that it carries the same allele as PI 163453, Danbaekkong, and Benning HP (**Supplementary Table S11**).

To quantify the Chr 20 fragment that was transferred from PI 163453 to Danbaekkong, 408 SNPs from the SoySNP50K SNP dataset distributed along the Chr 20 were used and it was observed that the PI 163453 fragment that was transferred to D76-8070 spans from 21 to 34.6 Mb and the D76-8070 fragment that was transferred to Danbaekkong starts at 2 Mb and ends at 36 Mb. Subsequently, a fragment from 0.2 to 37 Mb from Danbaekkong was transferred to Benning HP (**Supplementary Figure S4**). These results indicate that the high-protein allele is originally from PI 163453, and it was transferred to D76-8070 through the work of Hartwig. Then, D76-8070 was used in South Korea to develop Danbaekkong, which eventually returned to the United States and was used to develop the isogenic line Benning HP.

The haplotype of PI 163453 was also compared to the *G. soja* line PI 468916 used in the mapping study that identified the Chr 20 QTL (Diers et al., 1992). The comparison revealed that PI 163453 is only 43% similar to PI 468916 when considering all the SNPs in the 30–34 Mb window, but when comparing the sequence of the gene *Glyma.20g085100*, it was observed that PI 163463 is also missing the 321-bp fragment as PI 468916 (**Supplementary Table S11**, **Supplementary Figure S5**). These results indicate that although PI 163453 and PI 468916 are different at the haplotype level, they carry the same high-protein allele in *Glyma.20g085100*.

**Figure 7:**
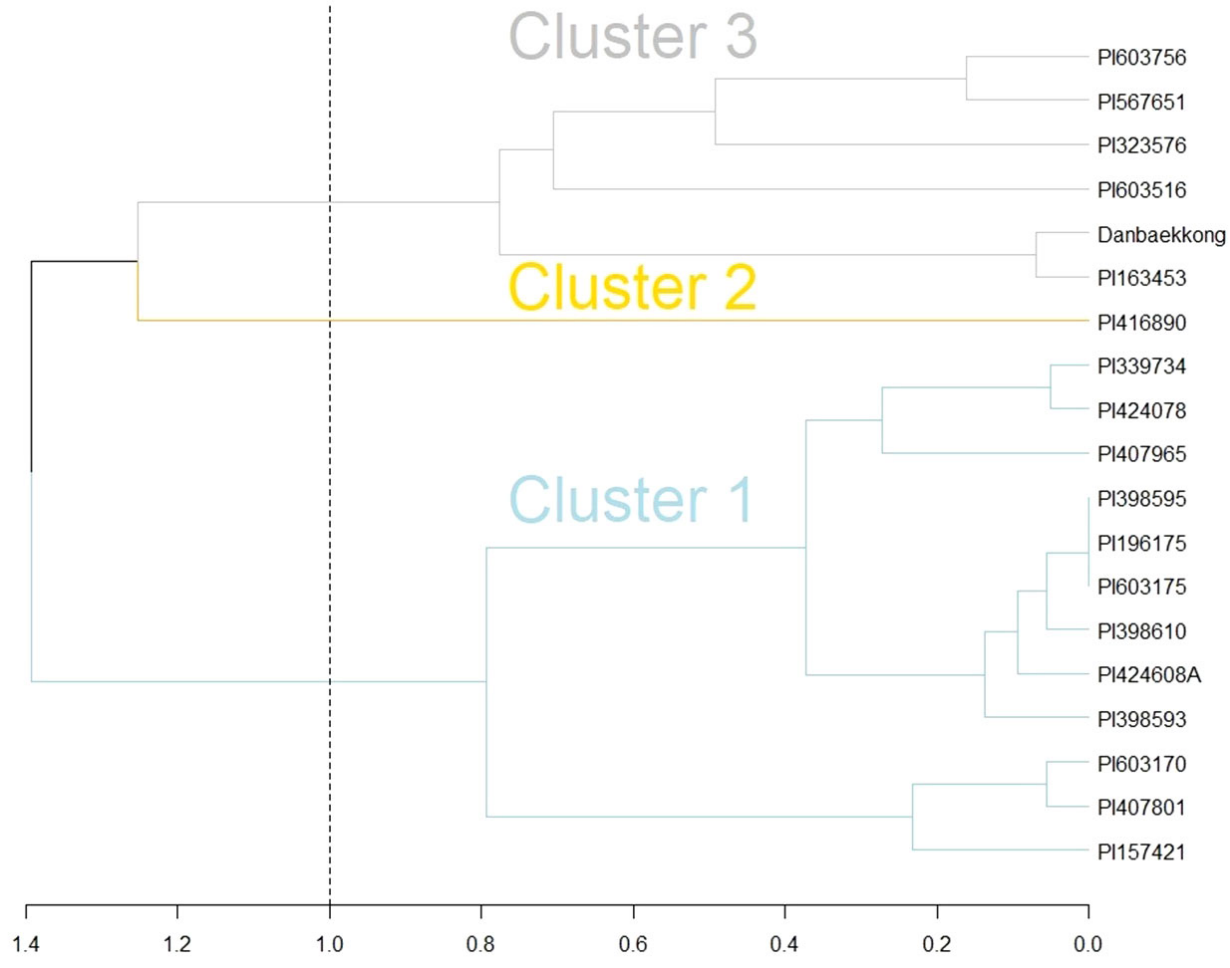
Comparison of PI 163453 and Danbaekkong haplotypes with the three introgression groups identified by Goettel et al. (2022) using hierarchical complete linkage cluster analysis. Analysis was based on 82 SNPs from the SoySNP50K at the Chr 20 QTL region between 29 and 34 Mb.

Goettel et al. (2022) indicated that the *Glyma.20g085100* high-protein allele was transferred from *G. soja* to *G. max* in three independent events likely during the process of domestication in East Asia. In the present research, it was demonstrated that the Danbaekkong high-protein allele came from the intentional introgression conducted by Edgar Hartwig where the *G. soja* PI 163453 was used as a grand parent to develop D76-8070 (Hartwig, 1990). Analyzing the haplotypes in the *Glyma.20g085100* region (Chr20, 29–34 Mb) revealed that both PI 163453 and Danbaekkong were grouped into cluster 3 identified by Goettel et al. (2022) (**Figure 7**). Cluster 3 is predominantly composed of the accessions from China except Danbaekkong that is a derived progeny from PI 163453.

### 4.2 Distribution of the Chr 20 high-protein allele among the soybean ancestors and *G. soja* lines

An analysis of the distribution of the high-protein allele was performed using 35 *G. max* that represents the diversity of the North American soybean cultivars (Gizlice et al., 1994). The results indicated that none of the 35 *G. max* ancestors carry the high-protein allele in *Glyma.20g085100.* However, three soybean ancestors, CNS (PI 548445), Arksoy (PI 548438), and Bilomi No. 3 (PI 240664), have protein content higher than 44% but do not carry the Chr 20 high-protein allele. CNS, Arksoy, and Bilomi No. 3 were originally collected in China, North Korea, and Philippines, respectively, and it is possible that these three accessions harbor protein QTLs in other genomic regions. To our knowledge, these ancestors have not been used in QTL mapping studies yet and they could reveal more information about the genetic control of protein in soybean.

Soybean lines with protein content reaching values of 47.2% have been developed (Wilcox and Cavins, 1995), and some lines have been released as cultivars in the United States in an effort to improve the seed composition, such as Protana with 43% protein (Probst et al., 1971), Prolina with 46% protein (Burton et al., 1999), and Prohio with 44.1% protein (Mian et al., 2008). More recently, soybean breeders focused on combining high yield and improved protein content and several breeding lines have been released. Chen et al. (2017) developed UA 5814HP as a new soybean cultivar with high seed protein content (45.5%) and yield comparable to elite checks. Pantalone and Smallwood (2018) released TN11-5102 as a high-yield and high-protein line with 42% protein. Shannon et al. (2022) developed S09-13185, with 44% protein content and Li et al. (2022) released G11-7013 with a protein content of 43.6%. Despite these efforts, the proportion of high-protein lines in North American germplasm is low. According to Patil et al. (2017), most soybean cultivars in the United States are fixed for the low-protein allele at the Chr 20 locus, and the introgression of the high-protein allele has the potential to improve the seed protein content in soybean cultivars in North America.

Goettel et al. (2022) analyzed a panel of 398 *G. max* (259 Cultivars and 139 Landraces) and 150 *G. soja* accessions from the USDA Soybean Germplasm Collection and observed that only 21 *G. max* lines had the Chr 20 high-protein allele. Of these 21 *G. max* lines that have the high-protein allele, 1 line was from India, 2 lines were from Japan, 4 lines were from China, and 14 lines were from South Korea, where Danbaekkong originated. Eight of the 14 Korean lines are cultivars with yellow seed coat, indicating that the Chr 20 high-protein allele has been selected and used in the development of soybean cultivars in Korean breeding programs. Lee et al. (2015) conducted a pedigree reconstruction of Korean soybean varieties and demonstrated that since 1913, soybean breeding programs have focused primarily on the improvement of seed protein composition for processing as soy food, such as soy sauce and tofu.

Differently from *G. max*, it was observed that all 79 *G. soja* from the USDA core collection analyzed carry the high-protein allele at *Glyma.20g085100*. When analyzing the sequence of additional 35 *G. soja* accessions, all of them also carry the high-protein allele. In a similar way, Goettel et al. (2022) analyzed a panel of 150 *G. soja* accessions and found that 147 lines had the high-protein allele confirmed. Owing to the widespread presence in *G. soja* of the high-protein allele in *Glyma.20g085100* and the low frequency in *G. max*, and the fact that *G. soja* is the closest ancestor to *G. max*, it is possible to infer that the high-protein allele is the original state of the gene. A few *G. soja* accessions present a low protein content, despite having the high-protein allele, especially the accessions PI 549046 and PI 597461B that showed a protein content lower than 40%. In fact, other studies have shown that on very few occasions, lines with the high-protein allele might have a low protein content, such as PI 407877B, PI 423954, and PI 424148 in Fliege et al. (2022). Goettel et al. (2022) indicated few wild soybean lines with a lower protein content but the overall mean protein of the *G. soja* with the high-protein allele was higher than the *G. max* with the low-protein allele. Vaughn et al. (2014) have also observed some cases where lines with the high-protein haplotype present a relatively low protein content. This is not fully understood; however, Kim et al. (2023) suggested that the protein content could be regulated by the interaction of multiple genes located at approximately 30 Mb on chromosome 20. Despite this, *Glyma.20g085100*, which is the gene targeted in this study, is likely the major gene in this regulation. The Chr 20 QTL has been shown to explain up to 55% of the variation (Warrington et al., 2015). Since it is not 100%, soybean genotypes can have relatively high or low protein through background segregation of these polygenic effects.

### 4.3 Effects of the Danbaekkong high-protein allele

A single marker analysis with the *Glyma.20g085100* marker was performed to understand the stability and effect of the gene across different genetic backgrounds. The analysis revealed that the high-protein allele inherited from Danbaekkong increased the protein by 3.3% on average (ranging from 2.6% to 3.7%) across all 10 populations tested in 2018 and 2019. The increase in protein content was also observed in the yield trials conducted in 2020 and 2021. In these trials, the high-protein allele had an average increase of 2.0% in the protein and only the population R12-514 × Benning HP did not show a significant increase in protein. This protein increase is similar to the estimate by Brzostowski et al. (2017), when the introgression of the Danbaekkong allele into two soybean lines caused an increase of 2% across four environments. The present results are close to the estimates by Warrington et al. (2015), where the author indicated a gain of 2.7% in protein with the Danbaekkong allele.

One of the well-known effects of the increase of protein content is the reduction of oil (Cober and Voldeng, 2000; Chung et al., 2003; Vaughn et al., 2014; Patil et al., 2018). According to Hanson et al. (1961), this relationship is dictated by a ratio of 2:1, in which the energy demanded to synthesize 2 protein units corresponds to 1 unit of oil. Other studies have shown that the protein-to-oil ratio is between 1.5 and 1.7 (Hartwig and Kilen, 1991; Chung et al., 2003). In the present research, it was observed that for every 1% increase in protein, there was a decrease of 0.55% in oil, representing a ratio of 1.8:1.

Several studies have indicated a negative relationship between protein and yield, with correlation values reaching up to −0.62 (Cober and Voldeng, 2000; Sebolt et al., 2000; Cunicelli et al., 2019). Overall, a negative correlation between these two traits appears to be common, but contrary to the omnipresent antagonist relationship between protein and oil, protein and yield do not have a consistent correlation when comparing multiple environments (Wilcox and Cavins, 1995; Prenger et al., 2019). In the present research, lines with the high-protein allele in general yield 313 kg ha^−1^ less (55 to 719 kg ha^−1^) than those with the low-protein allele within the same population. Brzostowski et al. (2017) found a yield reduction ranging from −273 to −558 kg ha^−1^ when introgressing the Danbaekkong allele into two soybean lines. In the same way, Goettel et al. (2022) indicated that the low-protein allele at *Glyma.20g085100* is associated with a yield increase of 150.3 kg ha^−1^. Despite the negative effect of the high-protein allele on yield, it was possible to identify lines carrying the high-protein allele (>43% protein) with comparable yield to the commercial checks (>95% yield). This shows that there is potential to couple high yield and high protein content with selection during breeding, and the negative association between protein and yield can be minimized.

An association between the presence of the high-protein allele in *Glyma.20g085100* and maturity was observed across different populations, where lines with the high-protein allele matured approximately 3.7 days earlier than their counterparts in the same population. Similar results were found by Prenger et al. (2019), where lines carrying the Danbaekkong allele matured earlier than those without the allele. The gene *Glyma.20g085100* is located 1.4 Mb upstream of the maturity locus E4 (Liu et al., 2008). Since Danbaekkong is an MG V cultivar, it is possible that it possesses the early maturity allele at the *E4* locus linked with the high-protein allele in a coupling phase. Therefore, the difference in maturity in lines with high protein derived from Danbaekkong is due to linkage between the high-protein QTL and the maturity gene *E4*.

To our knowledge, the present study was the first time a QTL for protein content in soybean has been fully assessed in a wide variety of genetic background simultaneously with several environments of yield trials, and its breeding history from *G. soja* to *G. max* has been described. This study complements and validates the findings of previous research about the role of *Glyma.20g085100* in determining the protein content in soybeans, providing more information about the effects and stability of the QTL, and confirming the value of its use to improve soybean seed composition.

## 5 Conclusions

In this research, a gene-specific marker, GSM1252, was designed for *Glyma.20g085100* and genotyping the bi-parental and multiparental populations confirmed the effectiveness of this marker as well as other flanking markers. This information can be useful resources for breeding programs to introgress the high-protein allele into elite lines. The analysis of the distribution of the *Glyma.20g085100* alleles revealed that the 35 *G. max* accessions that represent the genetic diversity of North American soybean cultivars have the low-protein allele, while the 79 *G. soja* accessions surveyed possess the high-protein allele. The analysis of the pedigree of Danbaekkong indicated that its high-protein allele was inherited from *G. soja* PI 163453, which is the same as the one from PI 468916. The Danbaekkong high-protein allele increased the protein content in all populations tested in 2018 and 2019 with an average of 3.3%, ranging from 2.6% in Benning HP × G10PR-56444R2 to a 3.7% increase in G13-6299 × Benning HP. The yield trials in 2020 and 2021, the allele increased the protein in 2% on average and was stable across multiple environments. It was observed that the increase in protein was accompanied by an overall decrease in oil and yield. However, it was possible to select breeding lines with the high-protein allele and yield comparable to elite checks, and this will enable the development of new cultivars with high protein content and high yield.

## Data availability statement

The genome sequencing data supporting the conclusions of this study have been deposited under the NCBI SRA database under BioProject PRJNA1031345.

## Author contributions

RS: Formal analysis, Data curation, Visualization, Writing – original draft. MARM: Investigation, Methodology, Resources, Writing – review & editing. JNV: Formal analysis, Software, Writing – review & editing. ZL: Conceptualization, Project administration, Methodology, Resources, Funding acquisition, Writing – review & editing.

## References

[B1] Bandillo N. Jarquin D. Song Q. Nelson R. Cregan P. Specht J.. (2015). A population structure and genome-wide association analysis on the USDA soybean germplasm collection. Plant Genome 8, 1–13. doi: 10.3835/plantgenome2015.04.0024 33228276

[B2] Bates D. Mächler M. Bolker B. M. Walker S. C. (2015). Fitting linear mixed-effects models using lme4. J. Stat. Software 67, 1–48. doi: 10.18637/jss.v067.i01

[B3] Bayer P. E. Yuan Y. Batley J. Nguyen H. T. Valliyodan B. Varshney R. K.. (2021). Sequencing the USDA core soybean collection reveals gene loss during domestication and breeding. Plant Genome 15, 1–12. doi: 10.1002/tpg2.20109 PMC12807262 34169673

[B4] Boerma H. R. Hussey R. S. Phillips D. V. Wood E. D. Rowan G. B. Finnerty S. L. (1997). Registration of ‘Benning’ Soybean. Crop Sci. 37, 1982–1982. doi: 10.2135/cropsci1997.0011183x003700060061x

[B5] Bolger A. M. Lohse M. Usadel B. (2014). Trimmomatic: A flexible trimmer for Illumina sequence data. Bioinformatics 30, 2114–2120. doi: 10.1093/bioinformatics/btu170 24695404 PMC4103590

[B6] Bolon Y. T. Joseph B. Cannon S. B. Graham M. A. Diers B. W. Farmer A. D.. (2010). Complementary genetic and genomic approaches help characterize the linkage group I seed protein QTL in soybean. BMC Plant Biol. 10, 1–24. doi: 10.1186/1471-2229-10-41 20199683 PMC2848761

[B7] Broman K. W. Wu H. Sen Ś. Churchill G. A. (2003). R/qtl: QTL mapping in experimental crosses. Bioinformatics 19, 889–890. doi: 10.1093/bioinformatics/btg112 12724300

[B8] Brumm T. J. Hurburgh C. R. (1990). Estimating the processed value of soybeans. J. Am. Oil Chem. Soc 67, 302–307. doi: 10.1007/BF02539680

[B9] Bryant C. (2020). Soybean production in Georgia. 1st ed (Athens: University of Georgia Cooperative Extension).

[B10] Brzostowski L. F. Pruski T. I. Specht J. E. Diers B. W. (2017). Impact of seed protein alleles from three soybean sources on seed composition and agronomic traits. Theor. Appl. Genet. 130, 2315–2326. doi: 10.1007/s00122-017-2961-x 28795235

[B11] Burton J. W. Carter T. E. Wilson R. F. (1999). Registration of ` prolina ‘ Soybean. Crop Sci. 39, 1993–1994. doi: 10.2135/cropsci1999.0011183X003900010066x

[B12] Chen P. Florez-Palacios L. Orazaly M. Manjarrez-Sandoval P. Wu C. Rupe J. C.. (2017). Registration of ‘UA 5814HP’ Soybean with high yield and high seed-protein content. J. Plant Regist. 11, 116–120. doi: 10.3198/jpr2016.09.0046crc

[B13] Chung J. Babka H. L. Graef G. L. Staswick P. E. Lee D. J. Cregan P. B.. (2003). The seed protein, oil, and yield QTL on soybean linkage group I. Crop Sci. 43, 1053–1067. doi: 10.2135/cropsci2003.1053

[B14] Cingolani P. Platts A. Wang L. L. Coon M. Nguyen T. Wang L.. (2012). A program for annotating and predicting the effects of single nucleotide polymorphisms, SnpEff: SNPs in the genome of Drosophila melanogaster strain w1118; iso-2; iso-3. Fly 6, 80–92. doi: 10.4161/cam.20753 22728672 PMC3679285

[B15] Cober E. R. Voldeng H. D. (2000). Developing high-protein, high-yield soybean populations and lines. Crop Sci. 40, 39–42. doi: 10.2135/cropsci2000.40139x

[B16] Cunicelli M. J. Bhandari H. S. Chen P. Sams C. E. Mian M. A. R. Mozzoni L. A.. (2019). Effect of a mutant danbaekkong allele on soybean seed yield, protein, and oil concentration. J. Am. Oil Chem. Soc 96, 927–935. doi: 10.1002/aocs.12261

[B17] de Borja Reis A. F. Tamagno S. Moro Rosso L. H. Ortez O. A. Naeve S. Ciampitti I. A. (2020). Historical trend on seed amino acid concentration does not follow protein changes in soybeans. Sci. Rep. 10, 1–10. doi: 10.1038/s41598-020-74734-1 33077826 PMC7572510

[B18] Diers B. W. Keim P. Fehr W. R. Shoemaker R. C. (1992). RFLP analysis of soybean seed protein and oil content. Theor. Appl. Genet. 83, 608–612. doi: 10.1007/BF00226905 24202678

[B19] Edwards K. Johnstone C. Thompson C. (1991). A simple and rapid method for the preparation of plant genomic DNA for PCR analysis. Nucleic Acids Res. 19, 1349. doi: 10.1093/nar/19.6.1349 2030957 PMC333874

[B20] Fliege C. E. Ward R. A. Vogel P. Nguyen H. Quach T. Guo M.. (2022). Fine mapping and cloning of the major seed protein quantitative trait loci on soybean chromosome 20. Plant J. 110, 1–15. doi: 10.1111/tpj.15658 PMC9303569 34978122

[B21] Garin V. Malosetti M. van Eeuwijk F. (2020). Multi-parent multi-environment QTL analysis: an illustration with the EU-NAM Flint population. Theor. Appl. Genet. 133, 2627–2638. doi: 10.1007/s00122-020-03621-0 32518992 PMC7419492

[B22] Gizlice Z. Carter T. E. Burton J. W. (1994). Genetic base for North American public soybean cultivars released between 1947 and 1988. Crop Sci. 34, 1143–1151. doi: 10.2135/cropsci1994.0011183X003400050001x

[B23] Goettel W. Zhang H. Li Y. Qiao Z. Jiang H. Hou D.. (2022). POWR1 is a domestication gene pleiotropically regulating seed quality and yield in soybean. Nat. Commun. 13, 3051. doi: 10.1038/s41467-022-30314-7 35650185 PMC9160092

[B24] Hanson W. D. Leffel R. C. Howell R. W. (1961). Genetic analysis of energy production in the Soybean. Crop Sci. 1, 121–126. doi: 10.2135/cropsci1961.0011183X000100020011x

[B25] Hartwig E. E. (1990). Registration of soybean high-protein germplasm line ‘D76-8070.’. Crop Sci. 30, 764–765. doi: 10.2135/cropsci1990.0011183x003000030092x

[B26] Hartwig E. E. Kilen T. C. (1991). Yield and composition of soybean seed from parents with different protein, similar yield. Crop Sci. 31, 290–292. doi: 10.2135/cropsci1991.0011183x003100020011x

[B27] Hwang E. Y. Song Q. Jia G. Specht J. E. Hyten D. L. Costa J.. (2014). A genome-wide association study of seed protein and oil content in soybean. BMC Genomics 15, 1–12. doi: 10.1186/1471-2164-15-1 24382143 PMC3890527

[B28] Keim P. Olson T. C. Shoemaker R. C. (1988). A rapid protocol for isolating soybean DNA. Soybean Genet. Newsl. 15, 150–152.

[B29] Kim S. D. Hong E.-H. Kim Y.-H. Lee S.-H. Seong Y.-K. Park K.-Y.. (1996). A new high protein and good seed quality soybean variety “Danbaegkong”. RDA. J. Agri. Sci. 38, 228–232.

[B30] Kim W. J. Kang B. H. Moon C. Y. Kang S. Shin S. Chowdhury S.. (2023). Quantitative trait loci (QTL) analysis of seed protein and oil content in wild soybean (Glycine soja). Int. J. Mol. Sci. 24 (4), 4077. doi: 10.3390/ijms24044077 36835486 PMC9959443

[B31] La T. Large E. Taliercio E. Song Q. Gillman J. D. Xu D.. (2019). Characterization of select wild soybean accessions in the USDA germplasm collection for seed composition and agronomic traits. Crop Sci. 59, 233–251. doi: 10.2135/cropsci2017.08.0514

[B32] Langmead B. Salzberg S. L. (2012). Fast gapped-read alignment with Bowtie 2. Nat. Methods 9, 357–359. doi: 10.1038/nmeth.1923 22388286 PMC3322381

[B33] Lee C. Choi M. Kim H. Yun H. Lee B. Chung Y.. (2015). Soybean [Glycine max (L.) merrill]: importance as A crop and pedigree reconstruction of korean varieties. Plant Breed. Biotech. 3, 179–196. doi: 10.1016/S0828-282X(08)70684-6

[B34] Lee S. Van K. Sung M. Nelson R. LaMantia J. McHale L. K.. (2019). Genome-wide association study of seed protein, oil and amino acid contents in soybean from maturity groups I to IV. Theor. Appl. Genet. 132, 1639–1659. doi: 10.1007/s00122-019-03304-5 30806741 PMC6531425

[B35] Lestari P. Van K. Lee J. Kang Y. J. Lee S.-H. (2013). Gene divergence of homeologous regions associated with a major seed protein content QTL in soybean. Front. Plant Sci. 4. doi: 10.3389/fpls.2013.00176 PMC3672674 23761803

[B36] Li Z. Bachleda N. Wilson B. Wood E. D. Buck J. W. Carter T. E.. (2022). Registration of G11-7013 soybean germplasm with high meal protein and resistance to soybean cyst nematode, southern root-knot nematode, and stem canker. J. Plant Regist. 16, 430–437. doi: 10.1002/plr2.20204

[B37] Liu B. Kanazawa A. Matsumura H. Takahashi R. Harada K. Abe J. (2008). Genetic redundancy in soybean photoresponses associated with duplication of the phytochrome A gene. Genetics 180, 995–1007. doi: 10.1534/genetics.108.092742 18780733 PMC2567397

[B38] McKenna A. Hanna M. Banks E. Sivachenko A. Cibulskis K. Kernytsky A.. (2010). The Genome Analysis Toolkit: A MapReduce framework for analyzing next-generation DNA sequencing data. Genome Res. 20, 1297–1303. doi: 10.1101/gr.107524.110.20 20644199 PMC2928508

[B39] Mian M. A. R. Cooper R. L. Dorrance A. E. (2008). Registration of ‘Prohio’ Soybean. J. Plant Regist. 2, 208–210. doi: 10.3198/jpr2007.09.0531crc

[B40] Muñoz F. Rodriguez L. S. (2020) BreedR: Statistical methods for forest genetic resources analysts. Available at: https://github.com/famuvie/breedR.

[B41] Naeve S. Miller-Garvin J. (2021). United States soybean quality - Annual Report 2021 (St. Paul: University of Minnesota).

[B42] Pantalone V. Smallwood C. (2018). Registration of ‘TN11-5102’ Soybean cultivar with high yield and high protein meal. J. Plant Regist. 12, 304–308. doi: 10.3198/jpr2017.10.0074crc

[B43] Patil G. Mian R. Vuong T. Pantalone V. Song Q. Chen P.. (2017). Molecular mapping and genomics of soybean seed protein: a review and perspective for the future. Theor. Appl. Genet. 130, 1975–1991. doi: 10.1007/s00122-017-2955-8 28801731 PMC5606949

[B44] Patil G. Vuong T. D. Kale S. Valliyodan B. Deshmukh R. Zhu C.. (2018). Dissecting genomic hotspots underlying seed protein, oil, and sucrose content in an interspecific mapping population of soybean using high-density linkage mapping. Plant Biotechnol. J. 16, 1939–1953. doi: 10.1111/pbi.12929 29618164 PMC6181215

[B45] Prenger E. M. Yates J. Mian M. A. R. Buckley B. Boerma H. R. Li Z. (2019). Introgression of a high protein allele into an elite soybean cultivar results in a high-protein near-isogenic line with yield parity. Crop Sci. 59, 2498–2508. doi: 10.2135/cropsci2018.12.0767

[B46] Probst A. H. Laviolette F. A. Athow K. L. Wilcox J. R. (1971). Registration of protana soybean. Crop Sci. 11, 312–312. doi: 10.2135/cropsci1971.0011183x001100020050x

[B47] Qi Z. Pan J. Han X. Qi H. Xin D. Li W.. (2016). Identification of major QTLs and epistatic interactions for seed protein concentration in soybean under multiple environments based on a high-density map. Mol. Breed. 36, 1–16. doi: 10.1007/s11032-016-0475-x

[B48] Robinson J. T. Thorvaldsdóttir H. Winckler W. Guttman M. Lander E. S. Getz G.. (2011). Integrative genomics viewer. Nat. Biotechnol. 29, 24–26. doi: 10.1038/nbt.1754 21221095 PMC3346182

[B49] Sebolt A. M. Shoemaker R. C. Diers B. W. (2000). Analysis of a quantitative trait locus allele from wild soybean that increases seed protein concentration in soybean. Crop Sci. 40, 1438–1444. doi: 10.2135/cropsci2000.4051438x

[B50] Shannon G. Chen P. Crisel M. Smothers S. Clubb M. Vieira C. C.. (2022). S09-13185: High-yield soybean germplasm with elevated protein concentration. J. Plant Regist. 16, 417–422. doi: 10.1002/plr2.20169

[B51] Song Q. Hyten D. L. Jia G. Quigley C. V. Fickus E. W. Nelson R. L.. (2013). Development and evaluation of soySNP50K, a high-density genotyping array for soybean. PloS One 8, 1–12. doi: 10.1371/journal.pone.0054985 PMC3555945 23372807

[B52] USDA (2023) Germplasm resources information network (GRIN) - national plant germplasm system. Available at: https://www.ars-grin.gov/ (Accessed March 7, 2023).

[B53] Valliyodan B. Brown A. V. Wang J. Patil G. Liu Y. Otyama P. I.. (2021). Genetic variation among 481 diverse soybean accessions, inferred from genomic re-sequencing. Sci. Data 8, 1–9. doi: 10.1038/s41597-021-00834-w 33558550 PMC7870887

[B54] Vaughn J. N. Nelson R. L. Song Q. Cregan P. B. Li Z. (2014). The genetic architecture of seed composition in soybean is refined by genome-wide association scans across multiple populations. G3 Genes. Genomes. Genet. 4, 2283–2294. doi: 10.1534/g3.114.013433 PMC4232554 25246241

[B55] Wang J. Mao L. Zeng Z. Yu X. Lian J. Feng J.. (2021). Genetic mapping high protein content QTL from soybean ‘Nanxiadou 25’ and candidate gene analysis. BMC Plant Biol. 21, 1–13. doi: 10.1186/s12870-021-03176-2 34416870 PMC8377855

[B56] Warrington C. V. Abdel-Haleem H. Hyten D. L. Cregan P. B. Orf J. H. Killam A. S.. (2015). QTL for seed protein and amino acids in the Benning × Danbaekkong soybean population. Theor. Appl. Genet. 128, 839–850. doi: 10.1007/s00122-015-2474-4 25673144

[B57] Warrington C. Abdel-Haleem H. Orf J. H. Killam A. S. Bajjalieh N. Li Z.. (2014). Resource allocation for selection of seed protein and amino acids in soybean. Crop Sci. 54, 963–970. doi: 10.2135/cropsci2013.12.0799

[B58] Wickham H. (2016). ggplot2. Elegant graphics for data analysis (New York: Springer-Verlag). doi: 10.1002/wics.147

[B59] Wilcox J. R. Cavins J. F. (1995). Backcrossing high seed protein to a soybean cultivar. Crop Sci. 35, 1036–1041. doi: 10.2135/cropsci1995.0011183X003500040019x

[B60] Zhou Z. Jiang Y. Wang Z. Gou Z. Lyu J. Li W.. (2015). Resequencing 302 wild and cultivated accessions identifies genes related to domestication and improvement in soybean. Nat. Biotechnol. 33, 408–414. doi: 10.1038/nbt.3096 25643055

