# Supplementary material for: Introgression of a Danbaekkong high-protein allele across different genetic backgrounds in soybean
Source: Front Plant Sci. 2023 Dec 20;14:1308731. doi: 10.3389/fpls.2023.1308731 (PMC10761420; doi:10.3389/fpls.2023.1308731)
Supplement: Supplementary file 1 [file DataSheet_1.docx]

# ***Supplementary Material***

## Supplementary Tables

Supplementary Table S1. Sequenced accessions information.

| Accession | Name | Type | Origin | Haplotype‡ | Protein (%) |
| --- | --- | --- | --- | --- | --- |
| PI 595645 | Benning | Cultivar | United States | - | 42.2 |
| PI 619083 | Danbaekkong | Cultivar | South Korea | + | 51.5 |
| PI 163453 | Quail Haven | *G. soja* | China | + | 44.7* |
| PI 398589 | KAS 390-3 | Landrace | South Korea | + | 55.1* |
| PI 408012 | KAERI 548-5 | Landrace | South Korea | + | 51.3* |
| PI 602447 | BARC-14 nodulated | Cultivar | United States | + | 47.6* |
| PI 468916† |  | *G. soja* | China | + | 44.0* |

† Sequencing data retrieved from https://www.ncbi.nlm.nih.gov/sra/?term=SRP045129

‡ Haplotype in the QTL region. (-) Low protein haplotype, (+) High protein haplotype.

*Values obtained from USDA GRIN at https://npgsweb.ars-grin.gov/gringlobal/search

Supplementary Table S2. Protein and oil content of progenitors and parents of the 10 populations.

| Line | Protein (%)† | Oil (%)† | Derived RILs |
| --- | --- | --- | --- |
| Danbaekkong‡ | 48.0 | 18.5 | NA |
| Benning HP | 45.6 | 19.0 | NA |
| Benning | 41.9 | 21.3 | NA |
| R12-514 | 43.6 | 19.8 | 119 |
| G11PR-56151R2 | 42.3 | 20.0 | 119 |
| Woodruff | 42.1 | 19.3 | 115 |
| G11PR-56238R2 | 41.4 | 19.8 | 109 |
| N10-711 | 41.3 | 19.3 | 114 |
| G10PR-56444R2 | 40.5 | 21.0 | 105 |
| N05-7432 | 39.9 | 20.9 | 121 |
| G13-6299 | 39.7 | 20.7 | 111 |
| N08-174 | 39.0 | 21.9 | 102 |
| N11-7046 | 37.8 | 21.4 | 100 |
| Total |  |  | 1115 |

† Protein and oil are averages of three years (2019, 2020, and 2021).

‡ Protein and oil are averages of two years (2017 and 2021).

Supplementary Table S3. Molecular markers used to fine map the Chr 20 QTL in the Benning × Danbaekkong population. Marker GSM1252 is TaqMan and all the other markers are KASP.

| Marker Name | Type | dbSNP ID | Wm82.a2.v1 Coordinate | Alleles | Reference |
| --- | --- | --- | --- | --- | --- |
| BARC_057077_14568 | SNP | ss107925764 | 14507618 | A_G | Warrington et al., 2015 |
| BARC_038157_09983 | SNP | ss107919194 | 24536596 | A_G | Warrington et al., 2015 |
| Satt239 | SSR | - | 25275083 | (AAT)22 | Warrington et al., 2015 |
| BARC_046722_12700 | SNP | ss107920968 | 25446707 | C_T | Warrington et al., 2015 |
| GSM0012 | SNP | - | 26201368 | G_T | Warrington et al., 2015 |
| Satt496 | SSR | - | 27664504 | (ATT)13 | Warrington et al., 2015 |
| GSM0449 | SNP | ss715637188 | 29813037 | T_C | New Marker |
| GSM0453 | SNP | ss715637217 | 30546685 | T_C | New Marker |
| GSM0454 | SNP | ss715637241 | 31195048 | G_T | New Marker |
| GSM1117 | SNP | - | 31666190 | T_A | New Marker |
| GSM1118 | SNP | - | 31707347 | T_C | New Marker |
| GSM1213 | SNP | - | 31775201 | A_G | New Marker |
| GSM1252 | Insertion | - | 31778817 | Insertion+/- | New Marker |
| GSM1121 | SNP | - | 31851276 | A_G | New Marker |
| GSM1122 | SNP | - | 31981547 | T_C | New Marker |
| GSM1123 | SNP | - | 32010590 | G_A | New Marker |
| GSM0455 | SNP | ss715637294 | 32282623 | T_C | New Marker |
| GSM0457 | SNP | ss715637315 | 32721955 | A_G | New Marker |
| GSM0450 | SNP | ss715637316 | 32752215 | T_C | New Marker |
| GSM1124 | SNP | - | 32865400 | C_A | New Marker |
| GSM0458 | SNP | ss715637323 | 32894943 | T_C | New Marker |
| GSM0627 | Insertion | - | 32958630 | - | New Marker |
| BARC_020713_04700 | SNP | ss107916725 | 34052339 | C_T | Warrington et al., 2015 |
| GSM0451 | SNP | ss715637431 | 34314607 | G_A | New Marker |
| Satt354 | SSR |  | 34569176 |  | Warrington et al., 2015 |
| Satt049 | SSR |  | 36842373 | (AAT)16 | Warrington et al., 2015 |

Supplementary Table S4. Markers used to dissect the Chr 20 QTL across the multi-parent populations. Marker GSM1252 is TaqMan and all the other markers are KASP.

| Marker Name | SNP ID | Position Wm82.a2 (bp) | Alleles | Favorable allele | Type | Genes |
| --- | --- | --- | --- | --- | --- | --- |
| GSM0449 | ss715637188 | 29813037 | T_C | C | Intergenic | *Glyma.20g080000*  *Glyma.20g080100* |
| GSM0453 | ss715637217 | 30546685 | T_C | C | Intergenic | *Glyma.20g081200*  *Glyma.20g081300* |
| GSM0454 | ss715637241 | 31195048 | G_T | T | Intergenic | *Glyma.20g082700*  *Glyma.20g082800* |
| GSM1117 | - | 31666190 | T_A | A | Intragenic | *Glyma.20g084500* |
| GSM1118 | - | 31707347 | T_C | C | Intragenic | *Glyma.20g084900* |
| GSM1213 | - | 31775201 | A_G | G | Intragenic | *Glyma.20g085100* |
| GSM1252 | - | 31778817 | Insertion+/- | - | Intragenic | *Glyma.20g085100* |
| GSM1121 | - | 31851276 | A_G | G | Intragenic | *Glyma.20g085500* |
| GSM1122 | - | 31981547 | T_C | C | Intragenic | *Glyma.20g085700* |
| GSM1123 | - | 32010590 | G_A | A | Intragenic | *Glyma.20g086000* |
| GSM0455 | ss715637294 | 32282623 | T_C | C | Intergenic | *Glyma.20g086700*  *Glyma.20g086800* |
| GSM0457 | ss715637315 | 32721955 | A_G | G | Intergenic | *Glyma.20g087500*  *Glyma.20g087600* |
| GSM0450 | ss715637316 | 32752215 | T_C | C | Intergenic | *Glyma.20g087600*  *Glyma.20g087700* |
| GSM1124 | - | 32865400 | C_A | A | Intragenic | *Glyma.20g087800* |
| GSM0458 | ss715637323 | 32894943 | T_C | C | Intergenic | *Glyma.20g087900*  *Glyma.20g088000* |
| GSM0627 | - | 32958630 | Insertion+/- | - | Intragenic | *Glyma.20g088500* |
| GSM0451 | ss715637431 | 34314607 | G_A | A | Intragenic | *Glyma.20g099900* |

Supplementary Table S5. Sequence of the markers developed to saturate the Chr 20 QTL. Marker GSM1252 is TaqMan and all other markers are KASP.

| Marker | Primer Sequence |
| --- | --- |
| GSM0449 | Fam: GAAGGTGACCAAGTTCATGCTTCGCTGACTCTGCCACTGc |
|  | Hex: GAAGGTCGGAGTCAACGGATTTCGCTGACTCTGCCACTGt |
|  | Rev: ATGGACGACGGAGTAAGCAT |
| GSM0450 | Fam: GAAGGTGACCAAGTTCATGCTGGAGCAGAAGAGGGGGATGc |
|  | Hex: GAAGGTCGGAGTCAACGGATTGGAGCAGAAGAGGGGGATGt |
|  | Rev: TGCTGGAACCTGGACGAT |
| GSM0451 | Fam: GAAGGTGACCAAGTTCATGCTCGTTGAGTGACTGAGAGCCCAa |
|  | Hex: GAAGGTCGGAGTCAACGGATTCGTTGAGTGACTGAGAGCCCAg |
|  | Rev: GCCCTATACTTACAGCAAAGAAGCA |
| GSM0453 | Fam: GAAGGTGACCAAGTTCATGCTGTCACCACTACCGACATTATCGc |
|  | Hex: GAAGGTCGGAGTCAACGGATTGTCACCACTACCGACATTATCGt |
|  | Rev: TTCAAACAAAGCCAGAAATGC |
| GSM0454 | Fam: GAAGGTGACCAAGTTCATGCTGAGCAAAAGAGAGGGAATCAg |
|  | Hex: GAAGGTCGGAGTCAACGGATTGAGCAAAAGAGAGGGAATCAt |
|  | Rev: GCTGACGAGAACTTGGGATG |
| GSM0455 | Fam: GAAGGTGACCAAGTTCATGCTCAACCTTCTTCTTCTACTTCTATCc |
|  | Hex: GAAGGTCGGAGTCAACGGATTCAACCTTCTTCTTCTACTTCTATCt |
|  | Rev: TGTTGCTCATGCTAAGCCATA |
| GSM0457 | Fam: GAAGGTGACCAAGTTCATGCTTTGTGGCTATTGAGAGTAACa |
|  | Hex: GAAGGTCGGAGTCAACGGATTTTGTGGCTATTGAGAGTAACg |
|  | Rev: GCTACTGCTCTTTCTTCATTTACGC |
| GSM0458 | Fam: GAAGGTGACCAAGTTCATGCTACAATGGGTGAAGTGAAGc |
|  | Hex: GAAGGTCGGAGTCAACGGATTACAATGGGTGAAGTGAAGt |
|  | Rev: GTAACCAGCGAGTACATGACCAA |
| GSM0627 | Fam: GAAGGTGACCAAGTTCATGCTACAGATTTACACAGTACGTTAAGGACAGT |
|  | Hex: GAAGGTCGGAGTCAACGGATTCATTCATCACCCAAAAGTACGTTAAG |
|  | Rev: AGTGGTCAAGAGGAAAACTTGTGAA |
| GSM1117 | Fam: GAAGGTGACCAAGTTCATGCTCAGTTCCATTCAGATTTCACATTTGCa |
|  | Hex: GAAGGTCGGAGTCAACGGATTCAGTTCCATTCAGATTTCACATTTGCt |
|  | Rev: GCTTGTTAGTTTGTCATCCCTTTCAT |
| GSM1118 | Fam: GAAGGTGACCAAGTTCATGCTAACCGAAGAAGAGCCACCCAc |
|  | Hex: GAAGGTCGGAGTCAACGGATTCAACCGAAGAAGAGCCACCCAt |
|  | Rev: CTTTGTTGGTCCAGTTCTTCGCTATTAG |
| GSM1121 | Fam: GAAGGTGACCAAGTTCATGCTAGCACAAGGAGATCAAATTAAGAACCg |
|  | Hex: GAAGGTCGGAGTCAACGGATTTAAGCACAAGGAGATCAAATTAAGAACCa |
|  | Rev: TGAGTAGCTGTATAGTTCAAATTGCTTG |
| GSM1122 | Fam: GAAGGTGACCAAGTTCATGCTCCTCCAATTGCAGACGTAACACc |
|  | Hex: GAAGGTCGGAGTCAACGGATTCCTCCAATTGCAGACGTAACACt |
|  | Rev: TTCCCTTTCTAGGAATGAGGAAGAACTA |
| GSM1123 | Fam: GAAGGTGACCAAGTTCATGCTTTCTCTGAATCAACAACACAGGAAATTa |
|  | Hex: GAAGGTCGGAGTCAACGGATTTTCTCTGAATCAACAACACAGGAAATTg |
|  | Rev: CCACATACATGGGTGATGAGAATAAC |
| GSM1124 | Fam: GAAGGTGACCAAGTTCATGCTGGAAAAATAATCTAAGCCTCGGTCAa |
|  | Hex: GAAGGTCGGAGTCAACGGATTGGAAAAATAATCTAAGCCTCGGTCAc |
|  | Rev: GGTGGGAGTTGAGGTTAAGGG |
| GSM1213† | Fam: GAAGGTGACCAAGTTCATGCTCATTAACACTAAATATACATGATCGAGAc |
|  | Hex: GAAGGTCGGAGTCAACGGATTCATTAACACTAAATATACATGATCGAGAt |
|  | Rev: CACCATGTTGCAGGATGTTG |
| GSM1252‡ | Fwd: CCTTGTTTATGGCTCTCTCC |
|  | Rev: TGCATCAACCAAGCCTTAT |
|  | Probe FAM (INS-): GCGGCAAGCATACTGCATTTT |
|  | Probe VIC (INS+): CGGCAAGCATACAACAACAACA |

† Marker designed in reverse 3`→ 5`

‡ GSM1252 is a TaqMan marker targeting the 321 bp insertion in the gene *Glyma.20g085100.*

Supplementary Table S6. Protein and oil content of 1115 RILs with the high protein allele (HP) and RILs with the low protein allele (LP) (from 10 populations evaluated under field conditions in 2018 and 2019 in Athens, Georgia.

| Population ID | Pedigree | Protein (%) | | | Oil (%) | | |
| --- | --- | --- | --- | --- | --- | --- | --- |
|  |  | HP | LP | Difference | HP | LP | Difference |
| P1 | G13-6299 × Benning HP | 44.5 | 40.8 | 3.7*** | 19.7 | 21.4 | -1.6*** |
| P2 | Woodruff × Benning HP | 45.4 | 42.1 | 3.2*** | 19.3 | 21.2 | -1.9*** |
| P3 | N10-711 × Benning HP | 45.2 | 41.8 | 3.4*** | 19.1 | 21.1 | -2.0*** |
| P4 | N05-7432 × Benning HP | 44.3 | 40.7 | 3.6*** | 19.9 | 21.6 | -1.7*** |
| P5 | N11-7046 × Benning HP | 44.2 | 41.5 | 2.7*** | 19.5 | 21.2 | -1.8*** |
| P6 | N08-174 × Benning HP | 43.5 | 39.9 | 3.6*** | 20.1 | 22.0 | -1.9*** |
| P7 | R12-514 × Benning HP | 44.4 | 41.2 | 3.2*** | 19.9 | 21.6 | -1.7*** |
| P8 | Benning HP × G10PR-56444R2 | 44.0 | 41.4 | 2.6*** | 20.0 | 21.4 | -1.4*** |
| P9 | Benning HP × G11PR-56151R2 | 44.5 | 41.0 | 3.5*** | 19.9 | 21.8 | -1.9*** |
| P10 | Benning HP × G11PR-56238R2 | 44.4 | 41.2 | 3.1*** | 19.3 | 21.3 | -2.0*** |
| Average |  | 44.5 | 41.1 | 3.3*** | 19.7 | 21.5 | -1. 8*** |

*, **, *** Significant difference at the 0.05, 0.01 and 0.001 probability level in a t-test, respectively.

Supplementary Table S7. Comparison of lines with the high protein allele (HP) and lines with the low protein allele (LP) in each pedigree. One hundred and three Recombinant Inbred Lines (RILs) were evaluated in yield trials from five environments†.

| Population ID | Pedigree | N | | Yield  (kg ha^-1^) | | Oil  (%) | | Protein (%) | | Protein yield (kg ha^-1^) | | Difference (HP-LP) | | | |
| --- | --- | --- | --- | --- | --- | --- | --- | --- | --- | --- | --- | --- | --- | --- | --- |
|  |  | HP | LP | HP | LP | HP | LP | HP | LP | HP | LP | Yield (kg ha^-1^) | Oil  (%) | Protein  (%) | Protein yield (kg ha^-1^) |
| P1 | G13-6299 × Benning HP | 3 | 14 | 4764 | 5044 | 19.1 | 20.6 | 43.2 | 40.1 | 2060 | 2022 | -279 | -1.5 | 3.1 | 38 |
| P2 | Woodruff × Benning HP | 4 | 4 | 4691 | 4841 | 19.4 | 20.3 | 43.7 | 42.4 | 2051 | 2051 | -150 | -0.9 | 1.4 | 0 |
| P3 | N10-711 × Benning HP | 4 | 23 | 4855 | 4910 | 19.2 | 20.3 | 43.8 | 41.4 | 2125 | 2031 | -55 | -1.0 | 2.4 | 94 |
| P4 | N05-7432 × Benning HP | 8 | 2 | 4811 | 5529 | 19.9 | 20.5 | 42.2 | 40.7 | 2031 | 2249 | -719 | -0.6 | 1.5 | -218 |
| P5 | N11-7046 × Benning HP | 2 | 6 | 4767 | 5069 | 19 | 20.5 | 42.8 | 40.6 | 2042 | 2059 | -302 | -1.5 | 2.2 | -17 |
| P6 | N08-174 × Benning HP | 1 | 6 | 4934 | 5079 | 20.7 | 20.9 | 42.3 | 39.7 | 2086 | 2015 | -145 | -0.2 | 2.6 | 72 |
| P7 | R12-514 × Benning HP | 2 | 11 | 4566 | 4807 | 20.6 | 20.5 | 41.4 | 41.0 | 1892 | 1973 | -241 | 0.0 | 0.4 | -81 |
| P8 | Benning HP × G10PR-56444R2 | NA | 4 | NA | 5311 | NA | 20.8 | NA | 40.9 | NA | 2173 | NA | NA | NA | NA |
| P9 | Benning HP × G11PR-56151R2 | 2 | 4 | 4841 | 5305 | 18.8 | 20.4 | 44.0 | 41.0 | 2131 | 2175 | -464 | -1.6 | 3.0 | -44 |
| P10 | Benning HP × G11PR-56238R2 | 1 | 2 | 4796 | 5257 | 19.1 | 21.1 | 42.0 | 40.9 | 2016 | 2150 | -461 | -2.0 | 1.1 | -134 |
|  | Average |  |  | 4781 | 5115 | 19.5 | 20.6 | 42.8 | 40.9 | 2048 | 2080 | -313^**^ | -1.0^**^ | 2.0^**^ | -32.0^NS^ |

**Statistically different in the paired t-test, p<0.01

† Athens-2020, Plains-2020, Athens-2021, Plains-2021, Tifton-2021*.*

Supplementary Table S8. Performance of selected RILs across five environments†*.* Line performance was compared to the highest yielding check. HP indicates the presence of the high protein allele and LP is the low protein allele.

| Line ID | Pedigree | GSM1252 | Yield (kg ha^-1^) | % Check yield | Protein (%) | Protein yield  (kg ha^-1^) |
| --- | --- | --- | --- | --- | --- | --- |
| AGS 738RR | Commercial check | LP | 5160 | 100 | 38.91 | 2020 |
| G19-11395 | N05-7432 × Benning HP | LP | 5880 | 113.8 | 40.6 | 2380 |
| G19-2050R2 | Benning HP × G10PR-56444R2 | LP | 5810 | 112.6 | 41.2 | 2400 |
| G19-11112 | G13-6299 × Benning HP | LP | 5570 | 107.8 | 39.1 | 2170 |
| G19-2192R2 | Benning HP × G11PR-56151R2 | LP | 5540 | 107.3 | 41.1 | 2280 |
| G19-2308R2 | Benning HP × G11PR-56238R2 | LP | 5530 | 107.0 | 40.6 | 2250 |
| G19-11120 | G13-6299 × Benning HP | LP | 5450 | 105.5 | 40.7 | 2220 |
| G19-2003R2 | Benning HP × G10PR-56444R2 | LP | 5420 | 105.0 | 40.4 | 2200 |
| G19-11535 | N11-7046 × Benning HP | LP | 5420 | 104.9 | 42.3 | 2300 |
| G19-2115R2 | Benning HP × G11PR-56151R2 | LP | 5420 | 104.9 | 40.4 | 2190 |
| G19-11605 | N08-174 × Benning HP | LP | 5410 | 104.8 | 39.1 | 2110 |
| G19-11114 | G13-6299 × Benning HP | LP | 5410 | 104.7 | 39.8 | 2160 |
| G19-11029 | G13-6299 × Benning HP | LP | 5390 | 104.3 | 39.6 | 2140 |
| G19-11507 | N11-7046 × Benning HP | LP | 5380 | 104.2 | 40.6 | 2190 |
| G19-11257 | N10-711 × Benning HP | LP | 5370 | 104.0 | 42.7 | 2290 |
| G19-2229R2 | Benning HP × G11PR-56151R2 | LP | 5360 | 103.9 | 41.7 | 2240 |
| G19-11204 | Woodruff × Benning HP | LP | 5320 | 103.1 | 42.7 | 2280 |
| G19-11637 | N08-174 × Benning HP | LP | 5240 | 101.6 | 39.0 | 2050 |
| G19-11035 | G13-6299 × Benning HP | LP | 5210 | 100.8 | 39.6 | 2070 |
| G19-11462 | N05-7432 × Benning HP | LP | 5190 | 100.5 | 40.7 | 2110 |
| G19-11191 | Woodruff × Benning HP | HP | 5190 | 100.5 | 43.6 | 2280 |

† Athens-2020, Plains-2020, Athens-2021, Plains-2021, Tifton-2021*.*

Supplementary Table S9. Analysis of the presence of the 321 bp insertion in *Glyma.20g085100* in 35 *Glycine soja* accessions based on genome sequencing data.

| PI number/cultivar name | Species | MG | Origin | 321 bp insertion |
| --- | --- | --- | --- | --- |
| Benning | *G. max* | VII | United States | Yes |
| Danbaekkong | *G. max* | V | South Korea | No |
| PI 163453 | *G. soja* | VI | China | No |
| PI 468916 | *G. soja* | III | China | No |
| PI 378699A | *G. soja* | VII | Japan | No |
| PI 407038 | *G. soja* | V | Japan | No |
| PI 407085 | *G. soja* | VI | Japan | No |
| PI 407156 | *G. soja* | VI | Japan | No |
| PI 407175 | *G. soja* | IV | South Korea | No |
| PI 407179 | *G. soja* | V | South Korea | No |
| PI 407183 | *G. soja* | V | South Korea | No |
| PI 407191 | *G. soja* | V | South Korea | No |
| PI 407229 | *G. soja* | V | South Korea | No |
| PI 407231 | *G. soja* | V | South Korea | No |
| PI 407240 | *G. soja* | V | South Korea | No |
| PI 407262 | *G. soja* | VI | South Korea | No |
| PI 407270 | *G. soja* | VI | South Korea | No |
| PI 407287 | *G. soja* | V | Japan | No |
| PI 407307 | *G. soja* | VI | China | No |
| PI 407315 | *G. soja* | V | South Korea | No |
| PI 407318A | *G. soja* | V | South Korea | No |
| PI 424014 | *G. soja* | V | South Korea | No |
| PI 424070B | *G. soja* | V | South Korea | No |
| PI 424107A | *G. soja* | VI | South Korea | No |
| PI 483466 | *G. soja* | V | China | No |
| PI 507609 | *G. soja* | VI | Japan | No |
| PI 507615 | *G. soja* | VI | Japan | No |
| PI 507619B | *G. soja* | VI | Japan | No |
| PI 507624 | *G. soja* | VII | Japan | No |
| PI 507638 | *G. soja* | VI | Japan | No |
| PI 507667 | *G. soja* | VI | Japan | No |
| PI 507830B | *G. soja* | 0 | Russia | No |
| PI 507847 | *G. soja* | II | Russia | No |
| PI 532450 | *G. soja* | I | China | No |
| PI 549047 | *G. soja* | III | China | No |
| PI 562534 | *G. soja* | NA | South Korea | No |
| PI 562557 | *G. soja* | NA | South Korea | No |
| PI 562568 | *G. soja* | NA | South Korea | No |
| PI 639623A | *G. soja* | 0 | Russia | No |

Supplementary Table S10. Genetic similarity between Benning, Danbaekkong, PI 163453 and PI 468916 at the Chr 20 locus (30 – 34 Mb, Wm82.a2.v1) calculated with 6,353 SNPs.

| Line ID | Benning | Danbaekkong | PI 163453 | PI 468916 |
| --- | --- | --- | --- | --- |
| Benning | 1.00 |  |  |  |
| Danbaekkong | 0.53 | 1.00 |  |  |
| PI 163453 | 0.53 | 0.99 | 1.00 |  |
| PI 468916 | 0.46 | 0.49 | 0.49 | 1.00 |

Supplementary Table S11. Accessions in the USDA Germplasm Collection with the highest similarity to Danbaekkong and genotyping result with the marker GSM1252. HP is the high protein allele and LP is the low protein allele at *Glyma.20g085100*.

| Line Name | Similarity† |  | Species | Origin | GSM1252 | Protein (%)‡ | Oil (%)‡ |
| --- | --- | --- | --- | --- | --- | --- | --- |
| Danbaekkong | 1 |  | *G. max* | South Korea | HP | 45.43 | 18.99 |
| BARC-6 | 1 |  | *G. max* | United States | HP | 52.21 | 17.56 |
| BARC-14 nodulated | 0.99 |  | *G. max* | United States | HP | 48.21 | 18.31 |
| Qi du qing pi dou | 0.99 |  | *G. max* | China | HP | 46.04 | 17.53 |
| Kwangankong | 0.95 |  | *G. max* | South Korea | HP | 44.18 | 19.24 |
| D76-8070 | 0.95 |  | *G. max* | United States | HP | 49.29 | 18.07 |
| PI 163453 | 0.94 |  | *G. soja* | China | HP | 44.62 | 14.85 |
| Benning |  |  | *G. max* | United States | LP | 42.02 | 22.24 |
| Benning HP |  |  | *G. max* | United States | HP | 43.32 | 21.21 |

† Based on 92 SNPs from the SoySNP50K located between 30 and 34 Mb on chromosome 20.

‡ Protein and oil content evaluated in a sample of 200 seeds from plants grown in greenhouse in 2022

## Supplementary Figures


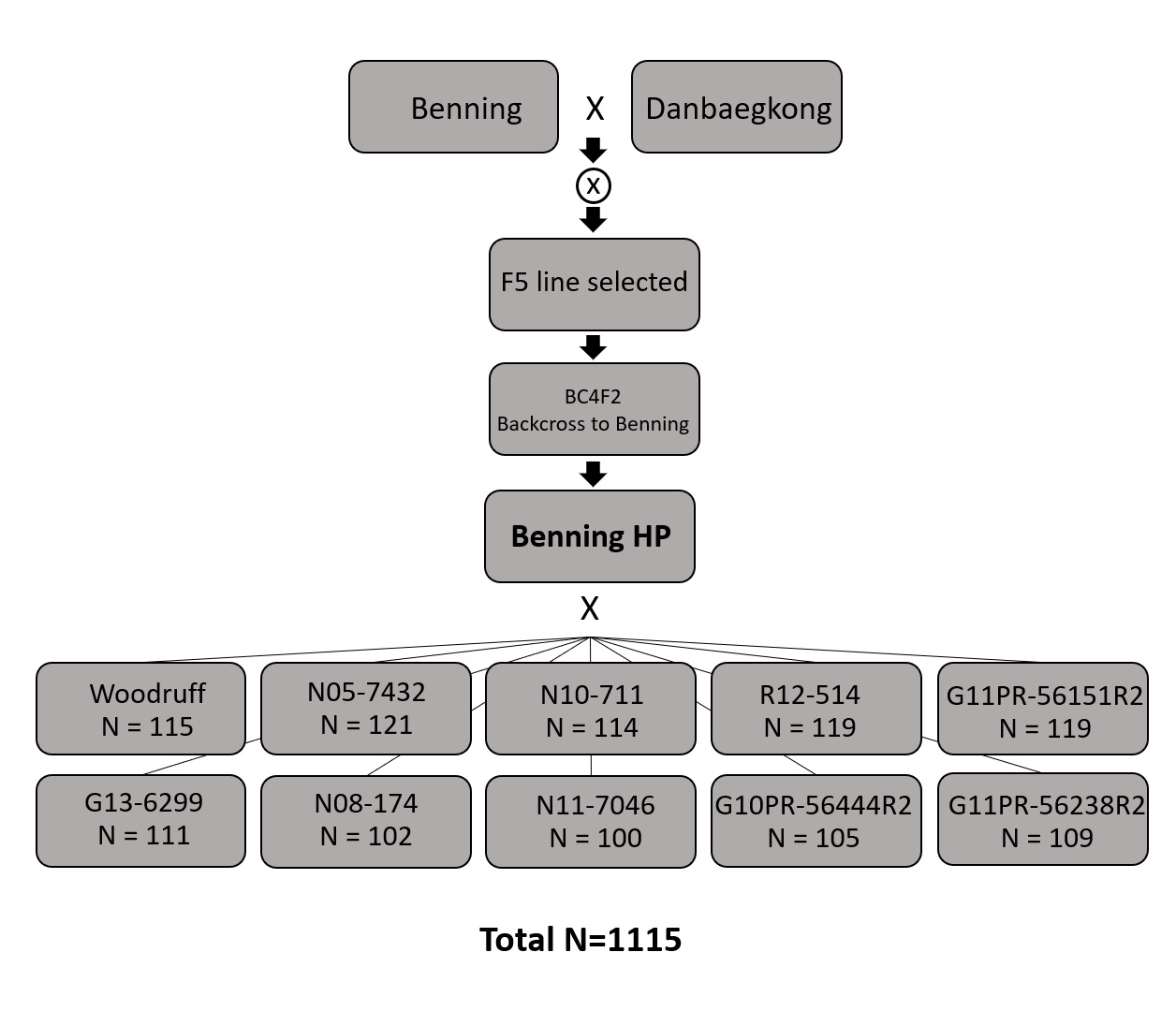


Supplementary Figure S1. Flowchart depicting the development of Benning HP and the derived RIL populations.


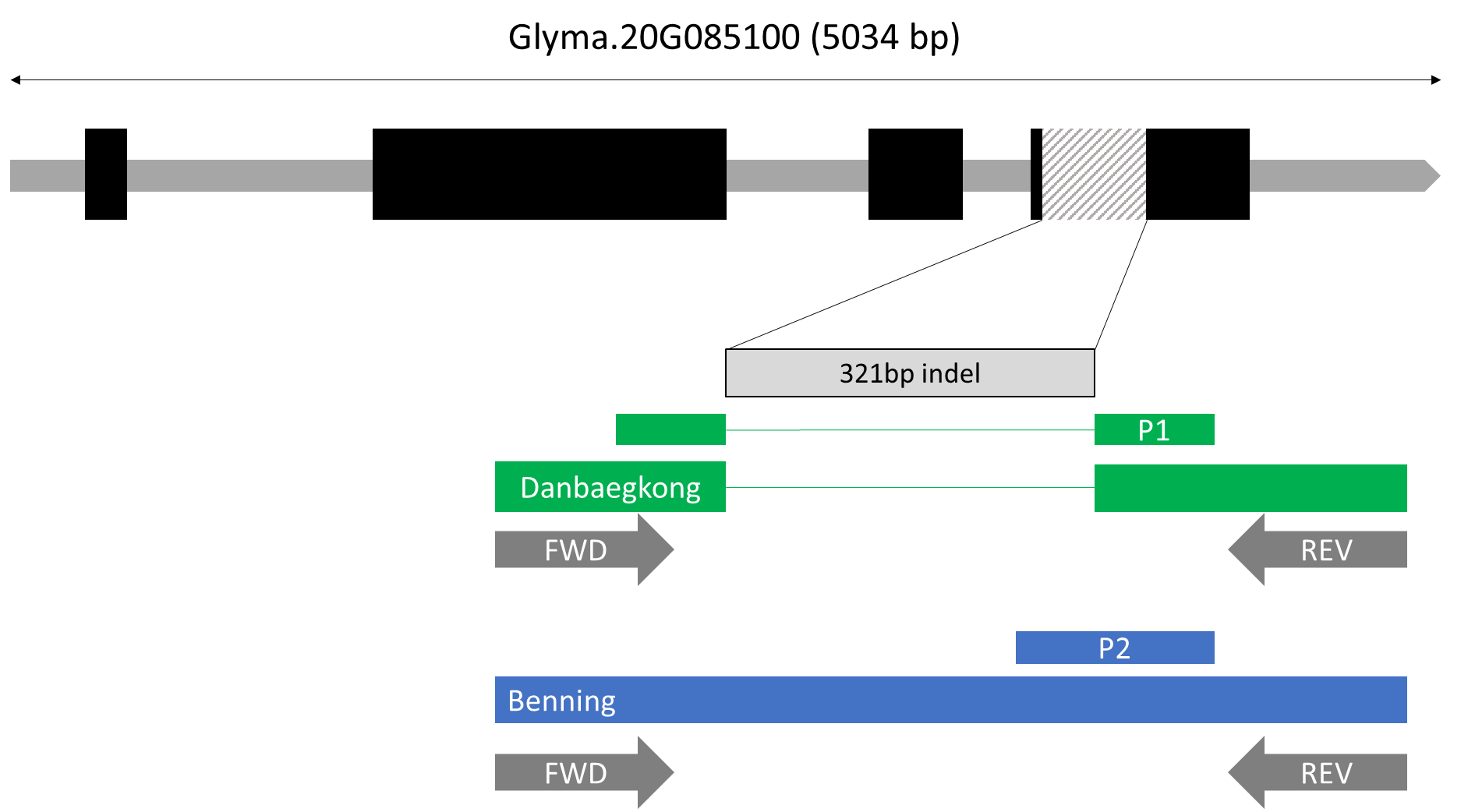


Supplementary Figure S2. Design of TaqMan marker GSM1252. P1 and P2 indicate the DNA probes and gray arrows indicate the primers.


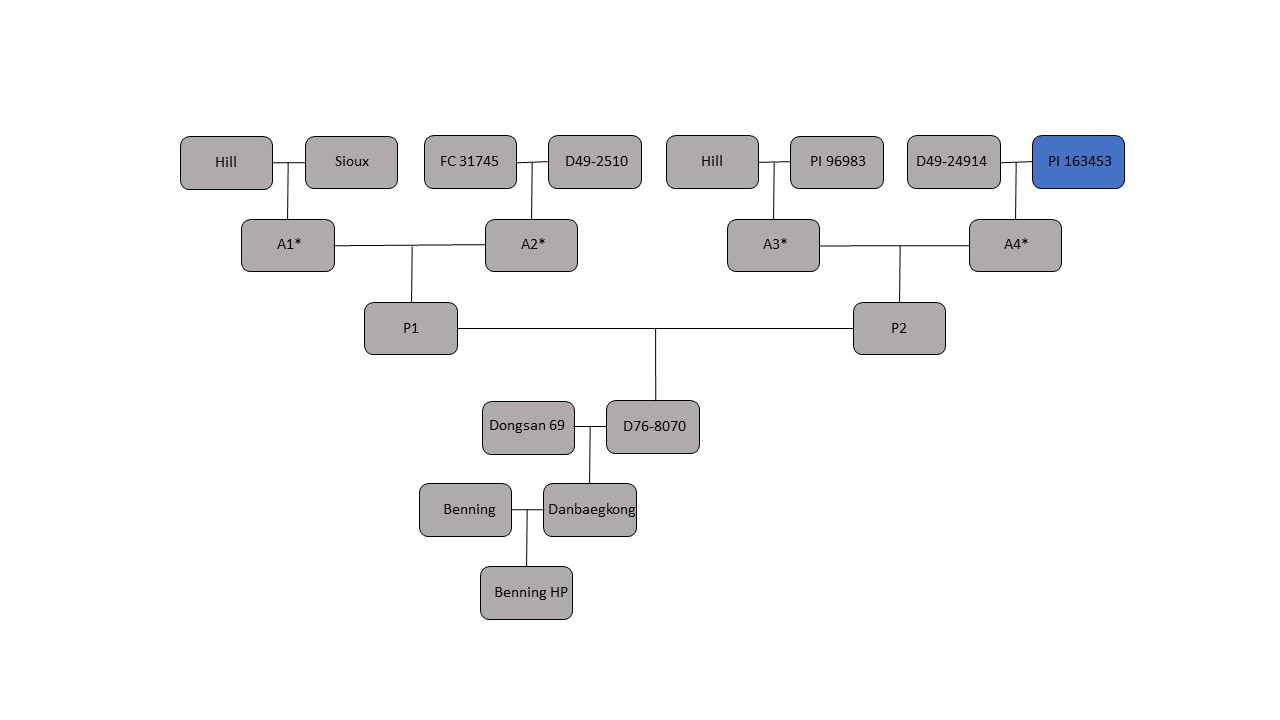


Supplementary Figure S3. Danbaekkong ancestry. * Indicates ancestor lines selected for resistance to bacterial pustule, shattering, and protein content higher than 45% (Hartwig, 1990).


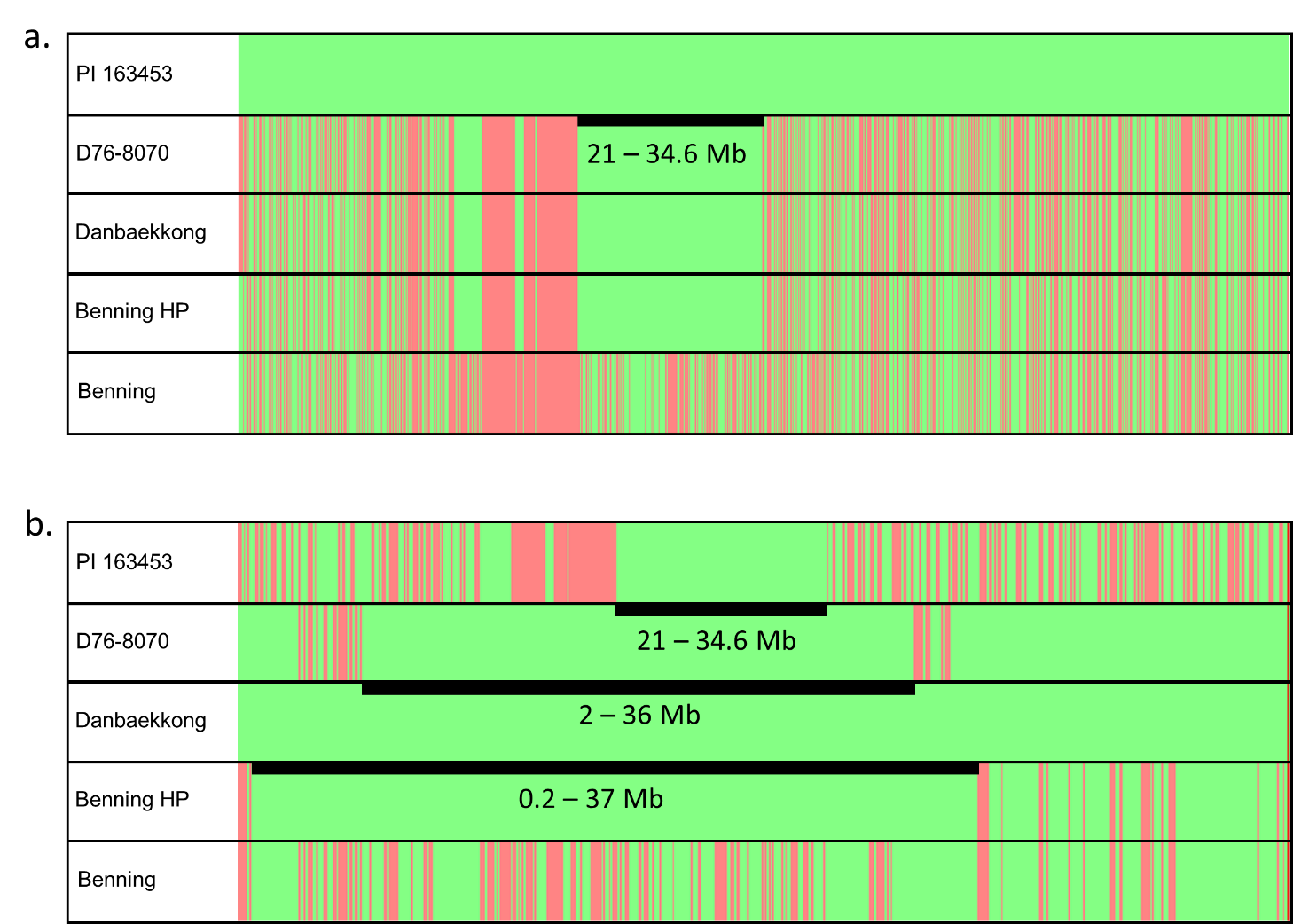


Supplementary Figure S4. PI 163453 Chr 20 fragment transferred to D76-8070 and subsequently to Danbaekkong and Benning HP. Comparison based on 1316 SNPs from the Soy50KSNP data. A) comparison using PI 163463 as the reference. B) comparison using Danbaekkong as the reference. Green indicates single nucleotide polymorphisms (SNPs) matching the reference genotype, and red denotes SNPs not matching the reference genotype.


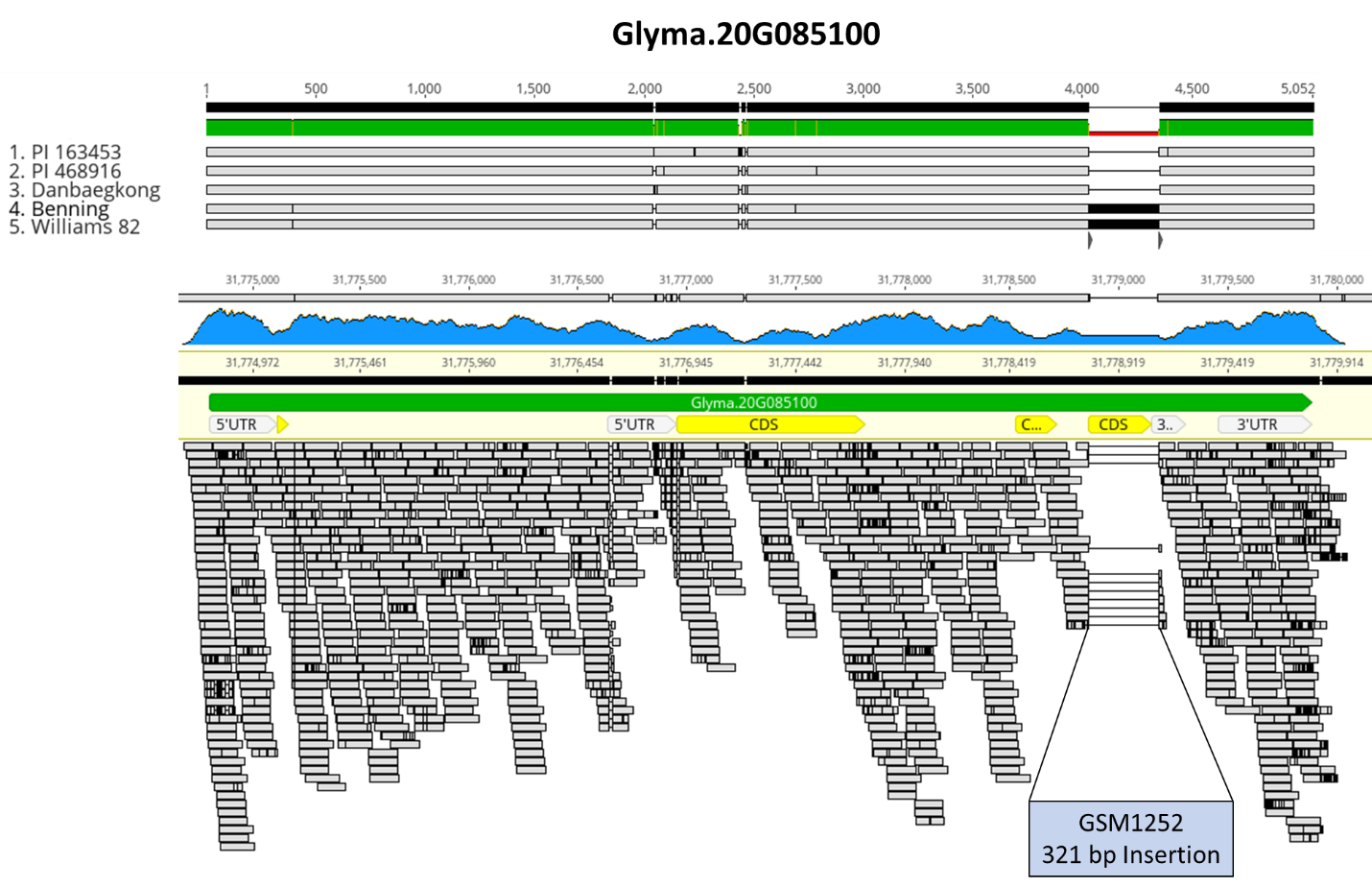


Supplementary Figure S5. Danbaekkong sequencing reads aligned to Williams 82.a2.v1 in the gene *Glyma.20g085100*. Sequence comparison of PI 163453, PI 468916, Danbaekkong, Benning, and Williams 82. The location of the TaqMan marker GSM1252 is indicated.


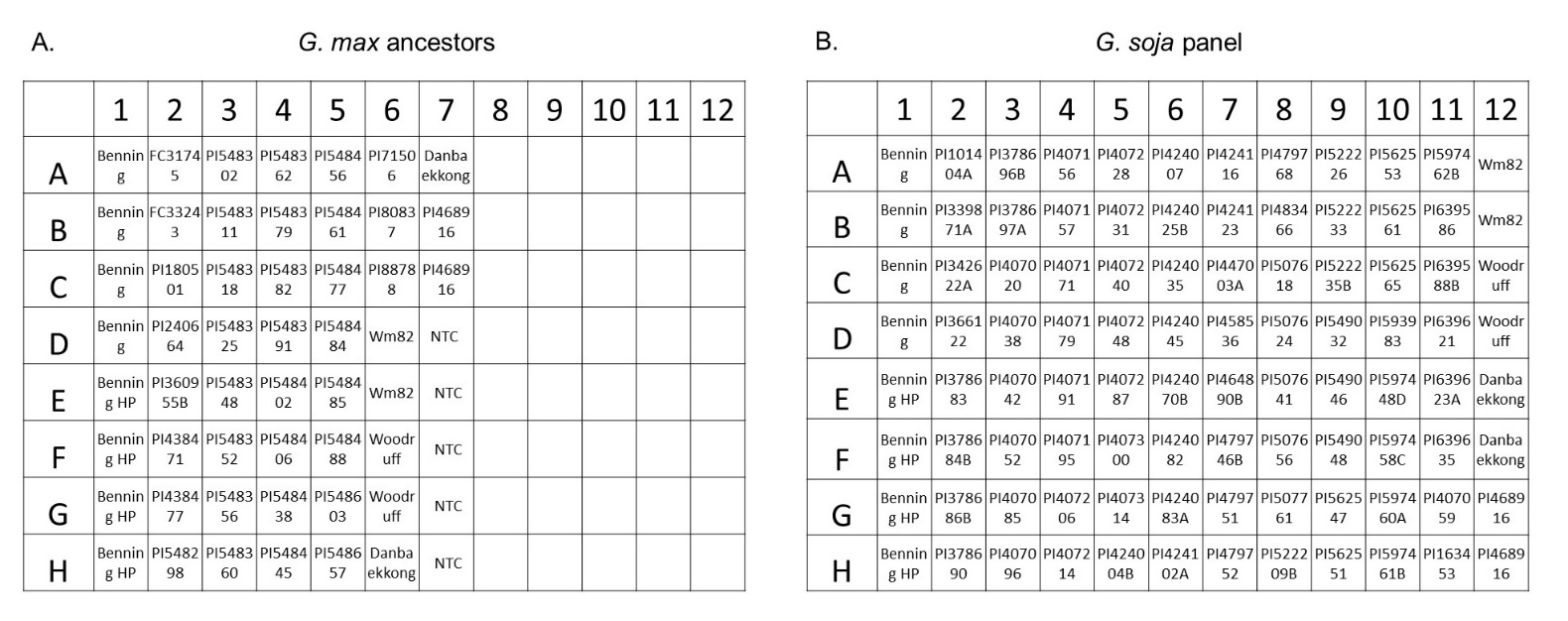


Supplemental Figure S6. A. DNA plate layout for the 35 North America *Glycine max* ancestors and B. 79 *Glycine soja* accessions genotyped with the gene specific TaqMan marker GSM1252. Benning and Benning HP were used as controls four times on each plate for the low protein (LP) and the high protein allele (HP), respectively. NTC stands for no template control. Additional control included were PI 163453 (HP), PI 468916 (HP), Danbaekkong (HP), Williams 82 (LP) and the cultivar Woodruff (LP).
